# Resveratrol blocks retrotransposition of LINE-1 through PPAR α and sirtuin-6

**DOI:** 10.1038/s41598-022-11761-0

**Published:** 2022-05-11

**Authors:** Noriyuki Okudaira, Yukihito Ishizaka, Mimi Tamamori-Adachi

**Affiliations:** 1 Department of Biochemistry, Teikyo University School of Medicine, 2-11-1, Kaga, Itabashi-ku, Tokyo, 173-8605 Japan; 2 Department of Intractable Diseases, National Center for Global Health and Medicine, 1-21-1 Toyama, Shinjuku-ku, Tokyo, 162-8655 Japan

**Keywords:** Biochemistry, Cell biology

## Abstract

The retroelement long interspersed element-1 (*LINE-1* or *L1)* comprises about 17% of the human genome. L1 retrotransposition is known to cause genomic instability and related disorders, and resveratrol suppresses this retrotransposition; however, the underlying mechanism is still not elucidated. Recent observations showed that low-molecular-weight compounds might induce *L1* retrotransposition through unknown mechanisms. This study aimed to determine polyphenol resveratrol (RV)’s effect on L1-RTP (retrotransposition) in somatic cells. Surprisingly, RV completely blocked L1-RTP. Experiments using the PPARα inhibitor GW6471 or siRNA-mediated PPARα depletion showed that RV-mediated L1-RTP’s inhibition depended on peroxisome proliferator-activated receptor α (PPARα). We demonstrated that RV inhibits p38 and cAMP response element binding protein phosphorylation, which are involved in MAPK signaling, and the L1-ORF1 protein’s chromatin recruitment. Furthermore, RV increased the expression of sirtuin-6 (SIRT6), which inhibited the activation of L1. The sirtuins family, SIRT1, SIRT6, and SIRT7, but not SIRT3, are involved in RV-mediated inhibition of L1-RTP. Overall, our findings suggest that RV directly modulates PPARα-mediated L1-RTP in somatic cells and that MAPK signaling interacts with SIRT6 closely and may play a role in preventing human diseases such as cancer.

## Introduction

Long interspersed element-1 (*LINE-1* or *L1*), a highly active autonomous and non-long terminal repeat retrotransposon, is the most abundant endogenous retroelement in humans and accounts for approximately 17% of the human genome^1–3^. L1 can retrotranspose to new genomic loci via a “copy and paste” mechanism. Notably, the L1 element can retrotranspose itself as well as other retroelements, such as Alu and SVA (SINE-VNTR-Alu: short interspersed element-variable number tandem repeat-Alu). A single human cell contains more than 5 × 10^5^ copies of L1, most of which are functionally inactive. However, 80–100 copies are capable of active retrotransposition. By analyzing the blood coagulation factor VIII gene in hemophilia A, it was found to be potentially capable of retrotransposistion in human genome^4^. Moreover, genetic mutation by L1 insertion has been reported, and a relationship with disease development is known. L1 insertions were reported in various human tumors, especially in cancer-related genes such as c-*myc* and *APC* in breast and colon carcinomas, respectively^5,6^. Recently, genome instability was a contributing factor to many age-related diseases. L1 drives IFN in senescent cells and developed age-related diseases^7^. L1-RTP is induced by disease development and environmental pollutants such as benzo[*a*]pyrene (B[*a*]P).

The L1 genomic sequence is ~ 6 kb in length and contains two open reading frames (ORF1 and ORF2), with molecular masses of ~ 40 and 150 kDa, respectively. ORF1 is a basic protein with a nucleic acid chaperone activity and is present within cytoplasmic ribonucleoprotein complexes or stress granules in the cytoplasm^8,9^. ORF2 is a multidomain protein with endonuclease and reverse transcriptase functions^10,11^. ORF1 and ORF2 perform L1-RTP (retrotransposition), which proceeds via three steps: transcription, reverse transcription, and insertion of the newly synthesized L1 DNA into the host genome via target-site primed reverse transcription^3,12,13^.

We recently reported that 6-formylindolo[3,2-b]carbazole (FICZ), a tryptophan photoproduct, acts as a potent inducer of L1-RTP^14^. We also found that L1-RTP is induced by 2-amino-1-methyl-6-phenylimidazo[4,5-*b*]pyridine (PhIP) and 2-amino-3,8-dimethyl-imidazo[4,5-*f*]quinoxaline (MeIQx), heterocyclic amines (HCAs) formed during the cooking of red meat as food-borne carcinogens, as well as benzo[*a*]pyrene (B[*a*]P), environmental pollutants^15,16^. Moreover, we reported that L1-RTP was induced during two-stage skin carcinogenesis initiated by 7,12-dimethylbenz[*a*]anthracene (DMBA) and 12-*O*-tetradecanoylphorbol-13-acetate^17^ and abused drugs including methamphetamine, cocaine, morphine, and fentanyl citrate^18,19^. We suggested that many carcinogenic and substance abuse drugs induced L1-RTP and genome instability, but we did not identify any compounds that inhibited L1-RTP.

Conversely, this study focuses on resveratrol (RV) (3,4′,5-trihydroxystilbene), a polyphenolic phytoalexin abundant in several plants and is also found in grapes, pine, and red wine^20^. Several studies have shown pleiotropic health benefits of RV, suggesting that it may prevent the progression of various age-related diseases^21–23^.

Our focus in this study was on peroxisome proliferator-activated receptor α (PPARα) as a ligand for RV, and sirtuins (SIRT) 6 are NAD^+^-dependent deacylases that play a crucial role in transcription and L1 resistant factor^24^. It has been reported that the expression of SIRT6 is increased when cells are exposed to RV. SIRT6 knockout mice drive inflammation and show strong L1 activation, and cells display excess genomic instability^25^. Moreover, SIRT7 mediated chromatin form regulation, Lamin A/C, and repression of L1 element transcription level^26^. Collectively, we took particular note of the sirtuins family and assessed the mechanism of L1-RTP inhibition by RV. We investigated if L1-RTP inhibition involves PPARa and SIRT6, which interact with SNF2H, a component of the SWI chromatin remodeling complex. SNF2H is a member of the SWI/SNF chromatin remodeling complex family known to regulate target genes’ transcription by altering chromatin structure. SIRT6 and SNF2H are chromatin remodeling factors involved in repair during DNA damage, suggesting that they are also involved in the aging process.

These observations suggest that human somatic cells are susceptible to genome instability by L1-RTP, but it is unclear whether L1-RTP inhibits cellular activity by altering genomic stability or if external stimuli mechanistically induce it. However, other studies have provided evidence that *L1* retrotransposition also occurs in somatic cells^27,28^*,* in which deregulation of the process may trigger various disease states owing to gene deletions. RV has been reported to inhibit L1 methylation and RTP, but the mechanism of this is unclear^29^. Therefore, we analyzed the L1 repressive mechanism of RV using the L1-RTP inducers we have previously reported. Elucidating the mechanisms that inhibit L1 function in RV could contribute to the aging process and the extension of a healthy life span.

## Results

### RV inhibited L1-RTP

**Figure 1:**
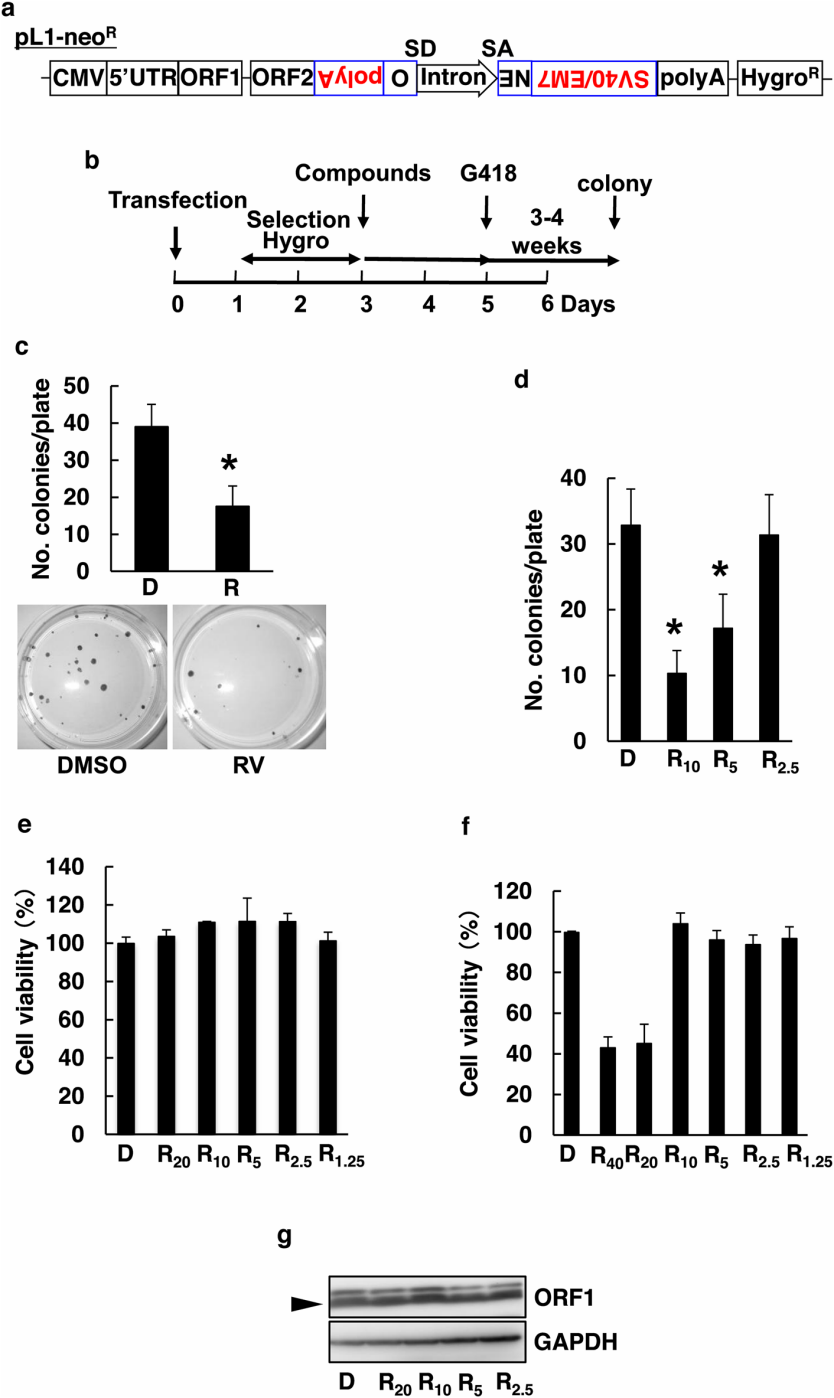
RV inhibits L1-RTP (**a**) Constructs used for assay of L1-RTP. (**b**) Experimental protocol of colony formation assay. HeLa cells or HuH-7 cells were transfected with pL1-Neo^R^ and selected for 2 days by hygromycin. Then, transfectants were exposed to compounds for 3 days and cultured for another 3–4 weeks in the presence of G418. (**c**) RV inhibited L1-RTP in HeLa cells. The transfection of pL1-Neo^R^ cells was incubated for 2 days with either 0.02% DMSO (lane 1) or RV at 20 μM (lanes 2). Colonies formed after treatment with DMSO (plate No. 1) or 20 μM RV (plate No. 2) are also shown. Colony numbers are presented as the mean ± standard deviation (SD). Asterisks indicate statistical significance (*p < 0.01 compared to DMSO). (**d**) RV inhibited L1-RTP in a dose-dependent manner. HuH-7 cells were incubated for 2 days with 0.02% DMSO (D) or RV at 10, 5, or 2.5 μM (R_10_, R_5_, R_2.5_). Colony numbers are presented as the mean ± SD. Asterisks indicate statistical significance (*p < 0.01 compared to DMSO). (**e**) No cytotoxic activity of RV on HeLa cells. Doses of RV sufficient to induce L1-RTP were not cytotoxic to HeLa cells. DMSO (0.1%) (D); RV (R_20_: 20 μM; R_10_: 10 μM; R_5_: 5 μM; R_2.5_: 2.5 μM; R_1.25_: 1.25 μM). The cells were treated for 2 days with RV. (**f**) Effects of RV on HuH-7 cells. Doses sufficient to induce L1-RTP (10 μM) were not cytotoxic to HuH-7 cells. DMSO at 0.04% (D); RV (R_40_: 40 μM; R_20_: 20 μM; R_10_: 10 μM; R_5_: 5 μM; R_2.5_: 2.5 μM; R_1.25_: 1.25 μM). The cells were treated for 2 days. Two independent experiments were performed, and representative results are shown. (**g**) No effects of RV on the expression of ORF1. RV was administered at doses sufficient to the level of ORF1 expression in HeLa cells. ORF1 is lower band (arrowhead). GAPDH was included as a loading control. DMSO (0.02%) control (D); 20 μM RV (R_20_); 10 μM RV (R_10_); 5 μM RV (R_5_); 2.5 μM RV (R_2.5_). The cells were treated for 24 h.

L1-RTP was detected by a colony formation assay, as described^14^. Briefly, HeLa cells were transfected with pCEP4/L1 mneol/ColE1 (Fig. 1a, pL1-Neo^R^) and selected for 2 days with hygromycin. RV for additional 2 days, cells were grown on neomycin for 3–4 weeks (Fig. 1b). The frequency of L1-RTP was evaluated by numbers of neomycin-resistant (Neo^R^) colonies. As shown in Fig. 1c and d, an initial experiment revealed that 5 ~ 20 μM RV significantly inhibited spontaneous L1-RTP. To exclude cytotoxic effects of RV, we examined plating efficiency. We observed that uM levels of RV, sufficient for inhibiting L1-RTP, were not toxic to HeLa cells (Fig. 1e) and HuH-7 cells (Fig. 1f). RV did not decrease the expression levels of the ORF1 protein (Fig. 1g, lower band). Based on these observations, we investigated whether μM levels of RV inhibit some retrotransposition steps activated by exogenous stimuli.

### RV inhibited L1-RTP by HCAs and FICZ

**Figure 2:**
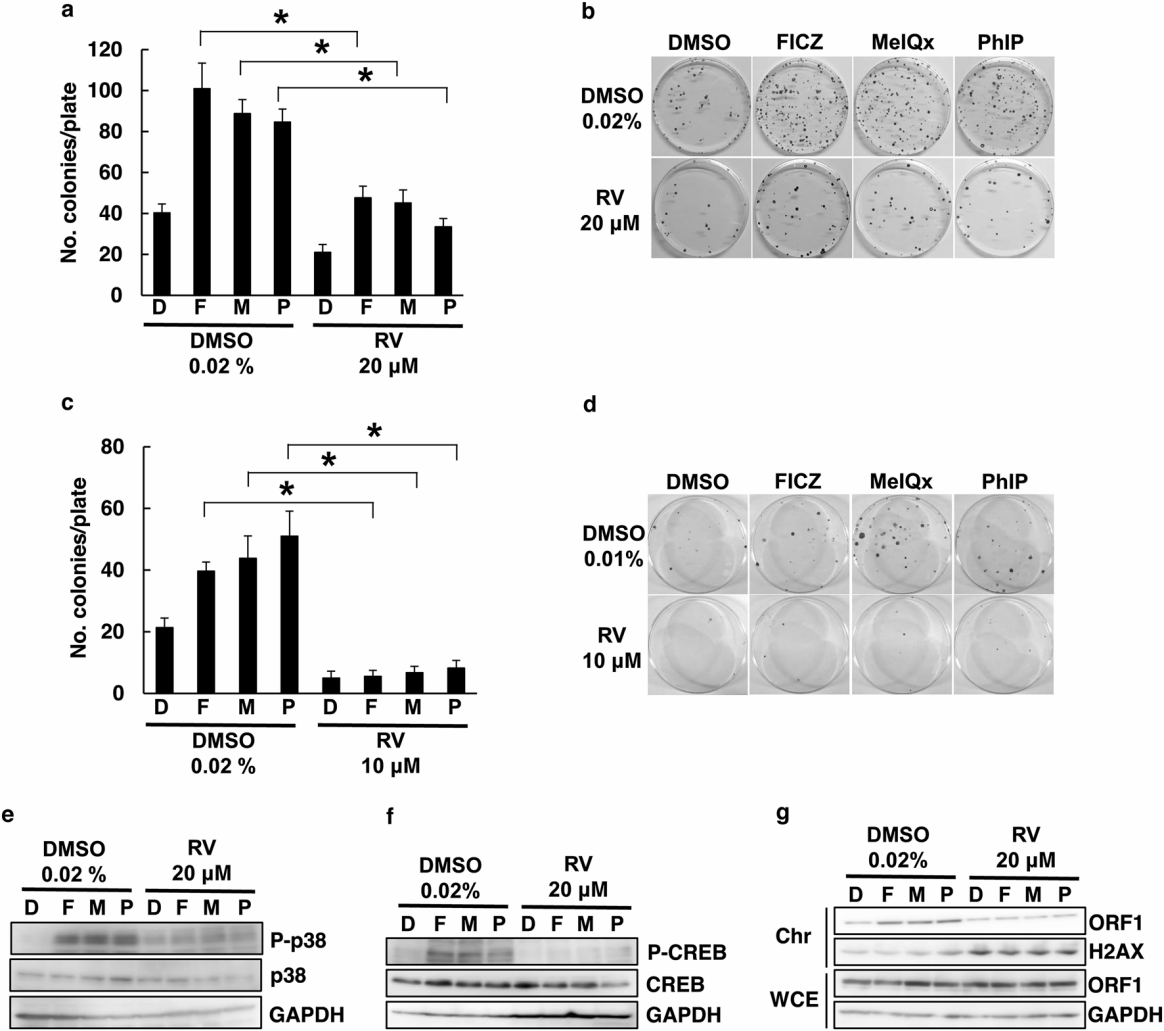
RV inhibits L1-RTP by environmental stimuli. (**a**) RV inhibits L1-RTP by HCAs and FICZ. HeLa cells were treated with 20 μM RV or 0.02% DMSO for 1 h prior to treatment with 0.02% DMSO (D), 10 nM FICZ (F), 18 μM MeIQx (M), or 18 μM PhIP (P). (*p < 0.01). (**b**) Colony formation assay of the effects of RV. (**c**, **d**) Colony formation assay of the effects of RV on L1-RTP. The same experiments of panels A and B were done using HuH-7 cells. (**e**, **f**) RV inhibited phosphorylation of p38 and CREB induced by environmental compounds. RV was added at 20 μM to the culture medium 30 min before the addition of 10 nM FICZ (F), 18 μM MeIQx (M), or 18 μM PhIP (P) for 30 min. (**g**) RV blocked chromatin recruitment of ORF1. HeLa cells were first transfected with pORF1-TAP. After 48 h, cells were treated with either 0.02% DMSO (lanes 1–4) or 20 μM RV (lanes 5–8) for 0.5 h, and then, environmental compounds were treated for another 24 h. DMSO (0.02%; D); lanes 1 and 5; 10 nM FICZ (F); lanes 2 and 6, 18 μM MeIQx (M); lanes 3 and 7, 18 μM PhIP; lanes 4 and 8. Whole cell lysate (WCE) and chromatin (Chr) fractions were prepared and analyzed. A representative result from two independent experiments is shown.

We recently discovered that environmental compounds that included FICZ, MeIQx, and PhIP act as potent inducers of L1-RTP^14,15^. We next examined the effect of RV on L1-RTP that is induced by these compounds. As shown in Fig. 2a and b, the induction of L1-RTP by FICZ, MeIQx, and PhIP was blocked by RV in HeLa cells. We also confirmed these experiments in the HuH-7 cells, a human hepatocellular carcinoma cell line (Fig. 2c,d). RV was previously reported to inhibit MAPK phosphorylation^30^. The p38 pathway and a transcription factor, cAMP response element binding protein (CREB), are present downstream of MAPK^31^. We have previously reported that FICZ, MeIQx, and PhIP induce L1-RTP by phosphorylating the p38 pathway and CREB. Therefore, we analyzed whether the phosphorylation of the p38 pathway and CREB by FICZ, MeIQx, and PhIP was inhibited by RV. To confirm this, we performed western blotting (WB) analysis, showing that RV markedly inhibited the phosphorylation of p38 and CREB by FICZ, MeIQx and PhIP (Fig. 2e,f). Mita P et al. reported that ORF1 and L1mRNA enter the nucleus during mitosis^32^. The ORF1 and L1-RTP import exhibits a strong cell cycle bias. FICZ, MeIQx, and PhIP induce the ORF1 nuclear transport^14,15^. This finding led us to hypothesize that RV blocked FICZ and HCAs induced chromatin recruitment of ORF1. In striking contrast, RV decreased ORF1 levels in the chromatin-rich fraction without apparent changes to the total amount of the protein. The FICZ- or HCA-induced chromatin recruitment of ORF1 was blocked by RV treatment (Fig. 2g). These data indicated that RV inhibited the interaction of L1 to chromatin via a mechanism involving the inactivation of p38-CREB signaling.

### SIRTs are involved in RV activity

**Figure 3:**
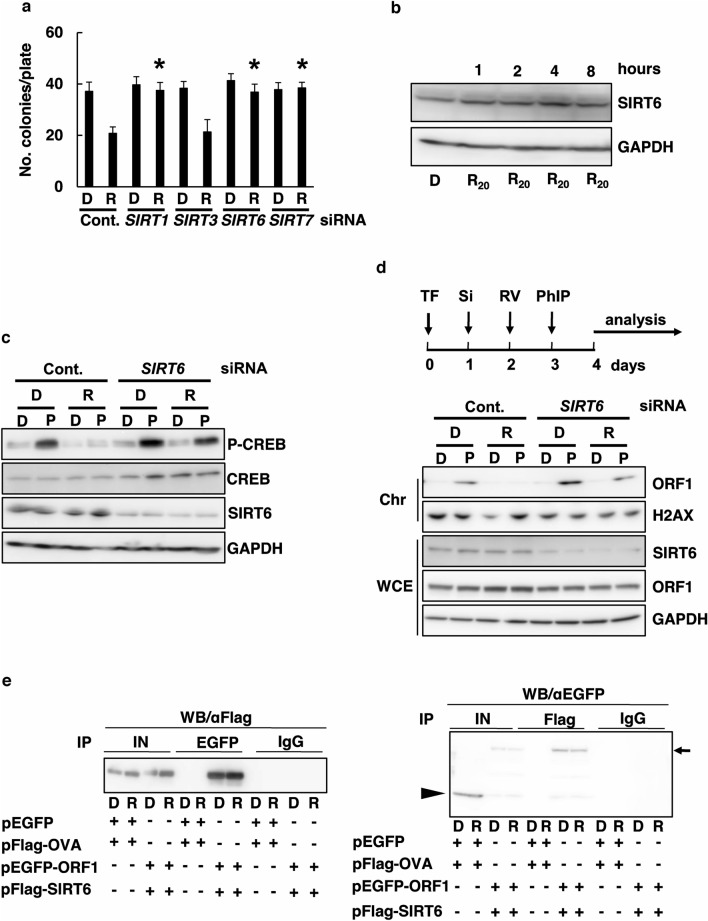
(**a**) SIRT1, SIRT6, and SIRT7 were required for RV-based inhibition of L1-RTP. A colony formation assay was performed for HeLa cells after introducing either control siRNA, *SIRT1*, SIRT3, *SIRT6*, and *SIRT7* siRNA. The cells were treated with DMSO (0.02%; D) or RV (20 μM; R) for 2 days. The mean numbers of colonies ± SD are shown. The effects of *SIRT1*,* SIRT3*,* SIRT6*, and* SIRT7* siRNAs were significant (*p < 0.01). Asterisks indicate statistical significance (*p* < 0.01 compared to control siRNA in RV treatment). (**b**) RV increases SIRT6 expression. A time course of RV on inducing SIRT6 expression in HeLa cells is shown. GAPDH was included as a loading control. DMSO (0.02%) control (D); 20 μM RV (R_20_; the cells were treated for 1, 2, 4, and 8 h). (**c**) Inhibitory effects of RV are linked with SIRT6. HeLa cells were transfected with either control siRNA at 10 nM or *SIRT6* siRNA-1 at 10 nM, respectively. Then, cells were treated with RV at 20 μM 30 min before the addition of 18 μM PhIP for 30 min. (**d**) Inhibition of chromatin recruitment of ORF1 by RV depends on SIRT6. According to a depicted protocol, experiments were done: HeLa cells were first transfected with pORF1-TAP on day 0, SIRT6 siRNAs on day one, and added with RV on day 2. On day 3, PhIP was added, and cells were subjected to analysis on day 4. DMSO of 0.02% (D) or 20 μM RV (R). DMSO (D), 18 μM PhIP (P). Chromatin (Chr) fractions and whole cell lysate (WCE) were prepared and analyzed. A representative result from two independent experiments is shown. (**e**) ORF1 associated with SIRT6. HEK293T cells were transfected with indicated constructs, and cell extracts were subjected to IP-WB analysis. IP of left panel was *α*EGFP → *α*Flag. IP of right panel was *α*Flag → *α*EGFP. Input (IN). 2nd antibody used True blot. Arrow and arrowhead indicate positions of EGFP-ORF1 and EGFP, respectively.

A recent study reported that many transcription factors and genes inhibited L1^33^. A previous study found that RV was involved with the sirtuin (SIRT) gene family^30^. SIRT1 and SIRT7 exist in the nucleus and nucleolus, respectively, while SIRT3 exists in the mitochondria. To further demonstrate SIRT1, SIRT3, SIRT6, and SIRT7 dependency, we described that two different siRNAs sequences (Table 1) against SIRT1, SIRT3, SIRT6, or SIRT7 efficiently suppressed their expression (Supplementary Fig. S1) and that both siRNAs abolished the RV-mediated L1-RTP inhibition caused by SIRT1, SIRT6, and SIRT7 (Fig. 3a). These data suggest that the siRNA abrogated endogenous SIRT1, SIRT6, and SIRT7 activity, thereby recovering L1-RTP. Moreover, we performed an analysis about SIRT3; as such, SIRT3 was not involved in RV-mediated L1-RTP inhibition. These data indicate that RV-based inhibition of L1-RTP depends on SIRT1, SIRT6, and SIRT7, but not on SIRT3. We performed experiments on SIRT6 for L1 activity^26^. We determined the effect of RV on SIRT6 expression. WB analysis showed that SIRT6 expression increased after RV treatment (Fig. 3b). We performed WB analysis, showing that SIRT6 siRNA markedly recovered the phosphorylation of CREB by RV inhibited PhIP (Fig. 3c), and the PhIP-induced chromatin recruitment of ORF1 was blocked by RV treatment (Fig. 3d). Downregulation of SIRT6 dissolves RV inhibited PhIP of L1-RTP (Supplementary Fig. S2). Moreover, a reciprocal experiment was performed using *αEGFP* followed by IP-WB analysis using *αFlag.* We determined whether ORF1 formed a complex with SIRT6. IP-WB analysis using expression vectors encoding EGFP-ORF1 and flag-tagged SIRT6 revealed that these two molecules were constitutively associated (Fig. 3e, left panel). By contrast, flag-tagged ovalbumin (OVA) was not associated with EGFP-ORF1 under the same conditions, indicating that the interaction of these molecules was not due to ORF1’s non-specific binding property. A reciprocal experiment confirmed the association of SIRT6 and ORF1 (Fig. 3e, right panel). Next, we confirmed whether SIRT1, 3, and 7 interact with ORF1 using the IP-WB analysis. Using HeLa cells, the SIRT gene group was investigated at the endogenous expression levels. The results showed that SIRT6 interacted with ORF1, but not SIRT1, 3, and 7. (Supplementary Fig. S3). These data indicate that ORF1 constitutively forms a complex with SIRT6, and RV promotes the association of SIRT6 with L1-ORF1, and thus, SIRT6 is involved in the RV-mediated L1-RTP inhibition.

**Table 1:** Summary of siRNAs used in the current study.

Genes	Nucleotide sequences (-5′ << 3′-)
***PPAR*α-1**	
Se	GUAUGGAAAUGGGUUUAUATT
AS	UAUAAACCCAUUUCCAUACGC
***PPAR*α-2**	
Se	GAUCAAGUGACAUUGCUAATT
AS	UUAGCAAUGUCACUUGAUCGT
***SIRT1*-1**	
Se	GUAAGACCAGUAGCACUAATT
AS	UUAGUGCUACUGGUCUUACTT
***SIRT1*-2**	
Se	GGCUUGAUGGUAAUCAGUATT
AS	UACUGAUUACCAUCAAGCCGC
***SIRT3*-1**	
Se	GCUCGGCAUCUGUUGGUUATT
AS	UAACCAACAGAUGCCGAGCTT
***SIRT3*-2**	
Se	GGAAGGCAUUACUCACUUATT
AS	UAAGUGAGUAAUGCCUUCCCT
***SIRT6*-1**	
Se	GUCUCCAGCUUAAACAGGATT
AS	UCCUGUUUAAGCUGGAGACCA
***SIRT6*-2**	
Se	GAAUGUGCCAAGUGUAAGATT
AS	UCUUACACUUGGCACAUUCTT
***SIRT7*-1**	
Se	GCCUGAAGGUUCUAAAGAATT
AS	UUCUUUAGAACCUUCAGGCTG
***SIRT7*-2**	
Se	GAAGCUACAUGGGAAGUGUTT
AS	ACACUUCCCAUGUAGCUUCAG
***SNF2H*-1**	
Se	GGAGAUACUUAGUAAUAGATT
AS	UCUAUUACUAAGUAUCUCCAA
***SNF2H*-2**	
Se	GGGCGAAAGUUCACUUAGATT
AS	UCUAAGUGAACUUUCGCCCAT

Nucleotide sequences of both sense and antisense strands were shown.

### SNF2H is involved in suppression by RV

**Figure 4:**
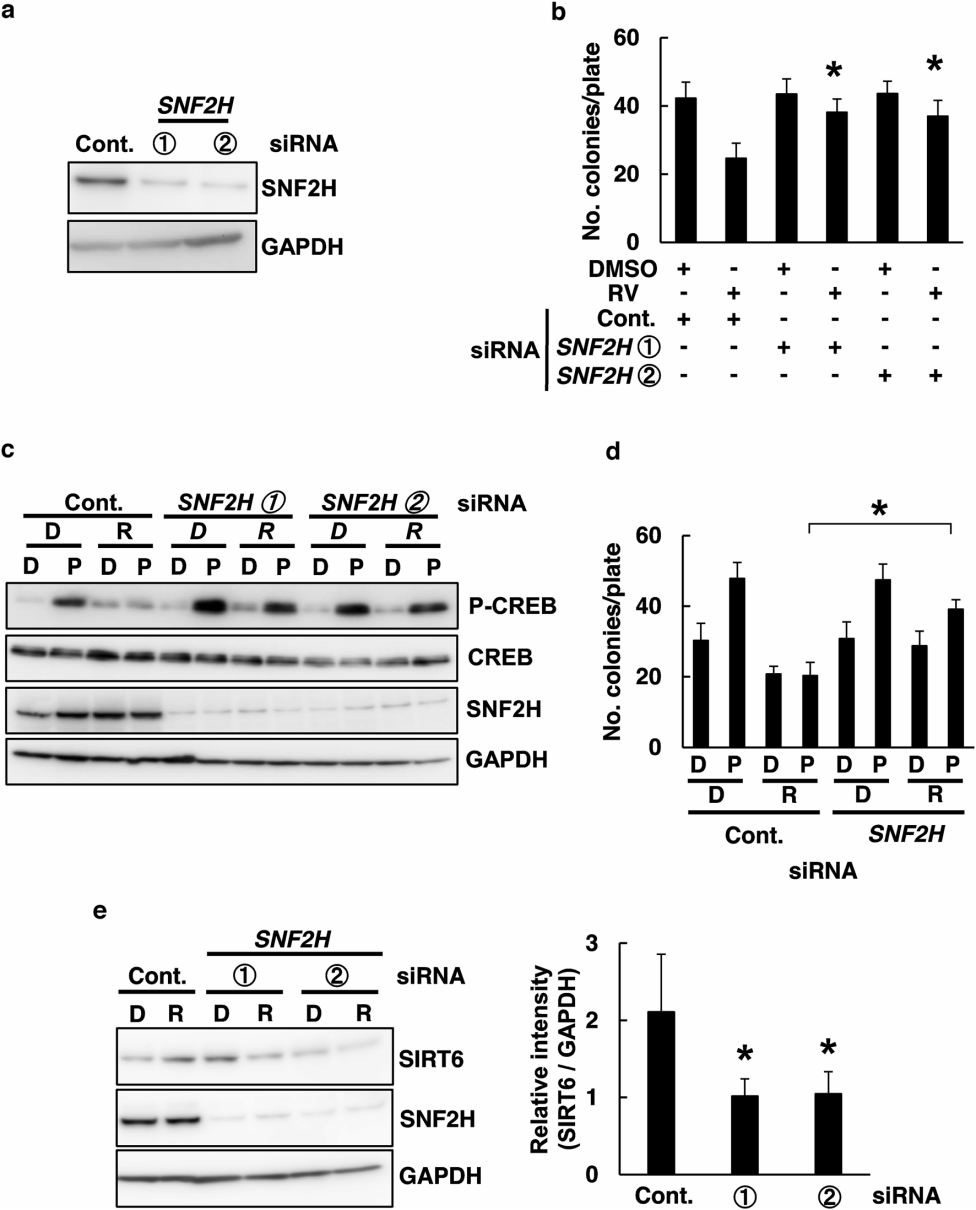
Inhibition of L1-RTP by RV depends on SNF2H. (**a**) Effects of *SNF2H* siRNA on the expression of the endogenous SNF2H protein. HeLa cells were transfected with pL1-Neo^R^ and siRNA. Cont.; Control siRNA (10 nM); ① and ②; SNF2H siRNA-1 or siRNA-2 (10 nM). (**b**) SNF2H was required to inhibit L1-RTP by RV. A colony formation assay was performed for HeLa cells after introducing either control siRNA or *SNF2H* siRNAs-1 or -2. The cells were treated with 0.02% DMSO or 20 μM RV for 2 days. The mean numbers of colonies ± SD are shown. The effects of *SNF2H* siRNAs were significant (*p < 0.01). (**c**) Downregulation of *SNF2H* the phosphorylation of CREB by PhIP. HeLa cells after introducing either control siRNA at 10 nM or *SNF2H* siRNA-1 or 2 at 10 nM. HeLa cells were first transfected with *SNF2H* siRNA, and then treated with RV (20 μM; R) to the culture medium 30 min before the addition of DMSO (0.02%; D) or PhIP (18 μM; P) for 30 min. (**d**) Downregulation of *SNF2H* blocked the inhibition of L1-RTP by RV. A colony formation assay was performed for HeLa cells after introducing either control siRNA or *SNF2H* siRNA-1. 0.02% DMSO: D, 18 μM PhIP: P, 20 μM RV: R. (**e**) RV-mediated SIRT6 expression depends on SNF2H. SNH2H is pivotal for RV-induced SIRT6 expression. WB analysis was performed after introducing either control siRNA or *SNF2H* siRNA-1,2 siRNA-1,2. The cells were treated with DMSO (0.02%; D) or RV (20 μM; R) for 2 days. The relative intensity ± SD is shown.

The ISWI family chromatin remodeling factor SNF2H and the SIRT6 for the immediate response to DNA double-strand breaks (DSBs). DSBs formation is associated with its chromatin incorporation regulated by SIRT6 and SNF2H. To examine whether SNF2H is involved in RV inhibited L1-RTP, and to further demonstrate this SNF2H dependency, we found that two *SNf2H* siRNAs (Table 1) efficiently suppressed the expression of *SNF2H* (Fig. 4a: data with siRNA-1 and siRNA-2 are shown) and that both siRNAs abolished RV-mediated L1-RTP inhibition (Fig. 4b). These data suggest that the siRNA abrogated endogenous SNF2H activity, thereby recovering L1-RTP. Moreover, we next examined the effect of SNF2H siRNA administration during L1-RTP induction of compounds. We performed WB analysis, showing that SNF2H siRNA markedly recovered CREB phosphorylation by RV inhibited PhIP (Fig. 4c). SIRT6 siRNA recovered the PhIP-induced L1-RTP by RV inhibited PhIP, respectively (Fig. 4d). RV-induced upregulation of SIRT6 expression is suppressed by siRNAs of *SNF2H* (Fig. 4e). These data indicate that SNF2H is involved in RV-mediated inhibition of L1-RTP.

### PPARα is involved in RV-suppression

**Figure 5:**
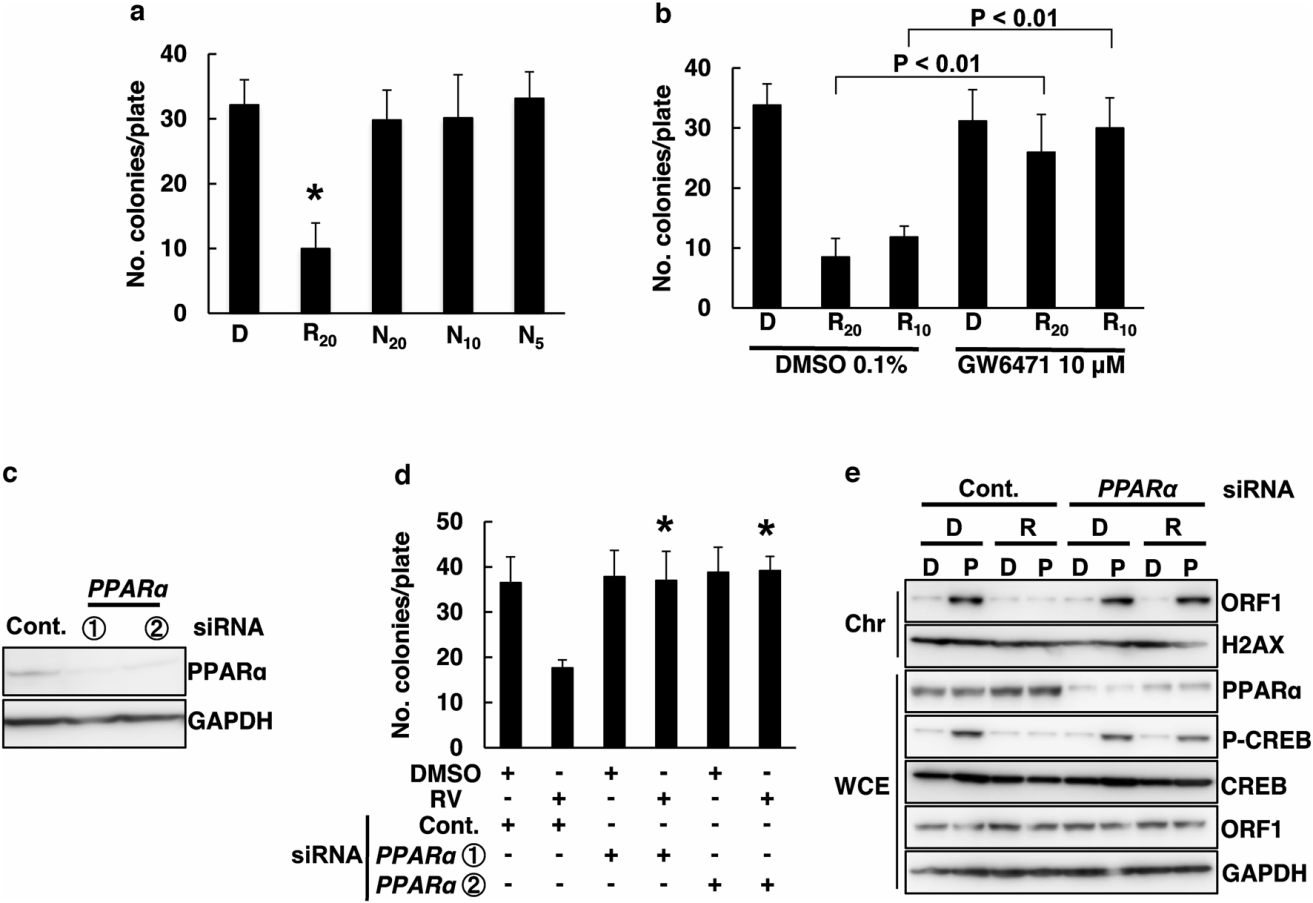
PPARα is involved in inhibitory effects of RV. (**a**) Inhibitory effects of RV inhibited on L1-RTP are independent of antioxidant effect. HeLa cells were incubated for 2 days with 20 μM RV or 5, 10, 20 mM NAC. DMSO (0.02%) control (D), 20 μM RV (R_20_), 20 mM NAC (N_20_), 10 mM NAC (N_10_), and 5 mM NAC (N_5_). Colony numbers are presented as the mean ± SD. (*p < 0.01). (**b**) Effect of a PPARα antagonist on the inhibitory effects of RV. HeLa cells treated with 10 μM RW6471 or 0.1% DMSO: D for 1 h before treatment with 20 μM RV: R_20_ or 10 μM RV: R_10_. (**c**) Effects of PPARα siRNA on the expression of the endogenous PPARα protein. Expression level of PPARα protein was examined in HeLa cells that had been transfected with pL1-Neo^R^ and *PPARα* siRNA. Cont., control siRNA (10 nM); ① and ②, PPARα siRNA-1 or siRNA-2 (10 nM), respectively. (**d**) PPARα was required to inhibit L1-RTP by RV. A colony formation assay was performed for HeLa cells after introducing either control siRNA or *PPARα* siRNA-1 or siRNA-2. The cells were treated with 0.02% DMSO (D) or 20 μM RV (R) for 2 days. The mean numbers of colonies ± SD are shown. The effects of *PPARα* siRNAs were significant (*p < 0.01). (**e**) Inhibition of chromatin recruitment of ORF1 by RV depends on *PPARα*. Experiments were done according to a depicted protocol by Fig. 3 D. DMSO (D) of 0.02% or 20 μM RV (R). DMSO (0.02%; D), 18 μM PhIP (P).

To determine the mechanism behind RV-mediated L1-RTP inhibition, we first assessed the involvement of RV’s antioxidant effect. According to a prior report, RV exerts a high antioxidant effect^34^. To confirm this, we performed L1-RTP analysis after treatment with RV or N-acetyl cysteine (NAC), an antioxidant compound serving as a positive control, which was found to have a minimal effect on L1-RTP (Fig. 5a). These observations indicated that RV-based inhibition of L1-RTP was independent of its antioxidant effect (Fig. 5a). Next, we evaluated the involvement of another possible factor. A recent study suggested that PPARα, a transcription factor associated with lipid metabolism, is an RV receptor^35,36^. Terasaki et al*.* previously reported that PPARα agonists increased L1 activity^37^. To test this, we first examined if the PPARα inhibitor GW6471 abolished the RV-mediated L1-RTP inhibition. Remarkably, we found that GW6471 completely blocked RV-based L1-RTP inhibition (Fig. 5b). To further demonstrate this PPARα dependency, we discovered that two *PPAR*α siRNAs (Table 1) efficiently suppressed the expression of *PPAR*α (Fig. 5c: data with siRNA-1 and siRNA-2 are shown) and that both siRNAs abolished RV-mediated L1-RTP inhibition (Fig. 5d: lanes 3–6). Next, we determined whether ORF1 is recruited to the chromatin fraction upon exposure of cells to *PPARα* siRNA, RV, and PhIP. When the ORF1-TAP transfectants were treated with RV and PhIP, the amount of ORF1 in the chromatin-rich fraction was recovered in *PPARα* siRNA lanes without apparent changes in the total amount of ORF1 (Fig. 5e).

### PPARα is associated with L1-ORF and SIRT6

**Figure 6:**
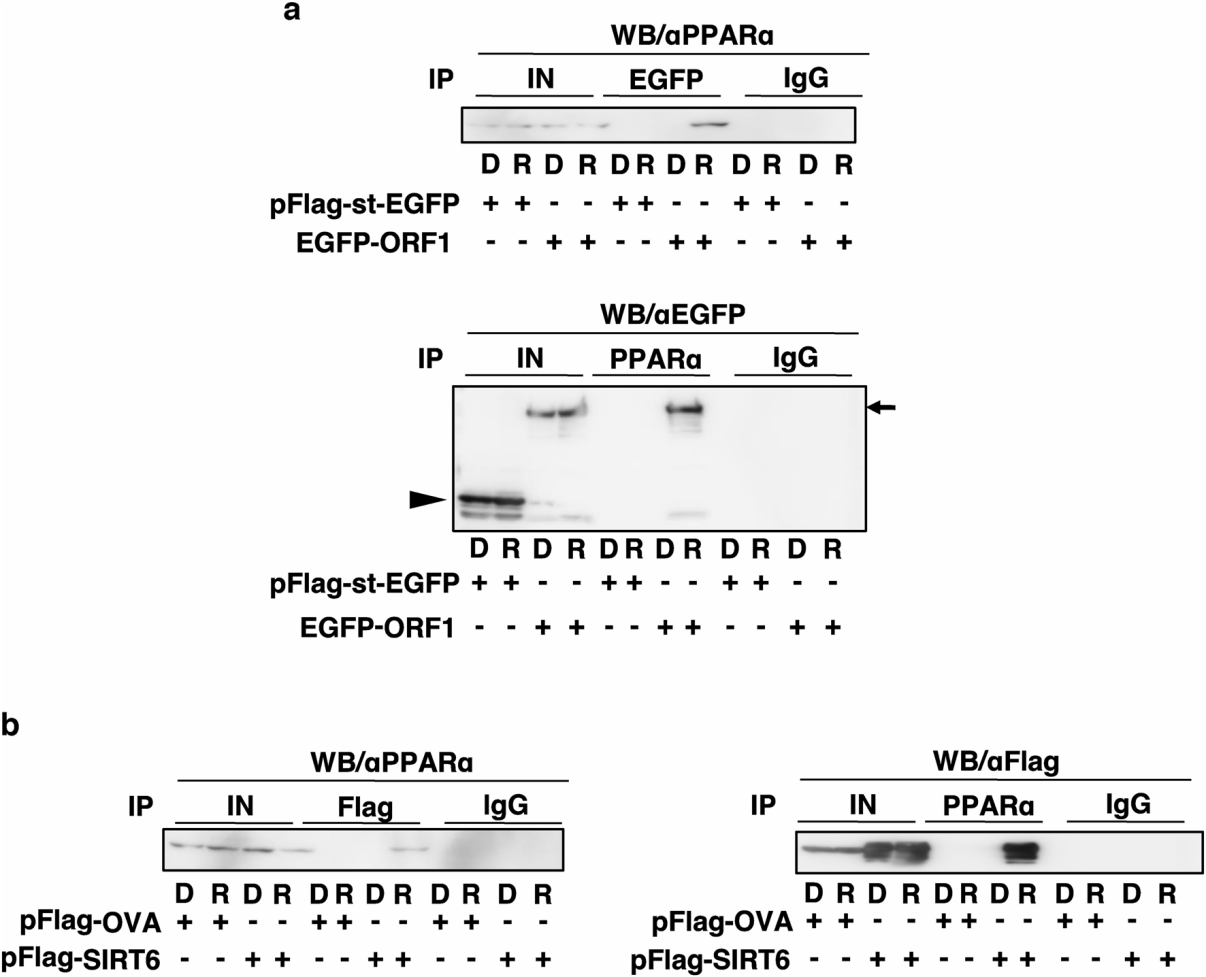
RV promotes the association of PPARα with L1-ORF1. (**a**) PPARα associated with ORF1 by RV. HEK293T cells were transfected with pFlag-st-EGFP or pEGFP-ORF1, and cell extracts were subjected to IP-WB. IP of the left panel was *α*EGFP → *α*PPARα. IP of the right panel was *α*PPARα → *α*EGFP. Input; IN. 2nd antibody used True blot. Arrow and arrowhead indicate positions of EGFP-ORF1 and Flag-st-EGFP, respectively. (**b**) PPARα associated with SIRT6 by RV. HEK293T cells were transfected with pFlag-OVA or pFlag-SIRT6, and cell extracts were IP-WB. IP of the left panel was *α*Flag → *α*PPARα. IP of the right panel was *α*PPARα → *α*Flag. The cells were treated with DMSO (0.02%; D) or RV (20 μM; R).

The chromatin recruitment of ORF1 experiments propounds its association with PPAR*α.* We transfected 293 T cells with pEGFP-ORF1 encoding a chimeric EGFP-ORF1 protein, treated them with RV, and performed IP-WB analysis to investigate this possibility. IP with αEGFP followed by αPPARα definitely revealed that ORF1 associated with PPARα upon RV treatment (Fig. 6a, upper panel). Moreover, a reciprocal experiment, in which IP was performed using αPPARα followed by WB analysis using αEGFP, detected the interaction of ORF1 and PPARα in RV treated cells (Fig. 6a, lower panel). Collectively, these data indicate that ORF1 associated with PPARα in response to the addition of RV inhibited L1-RTP. Moreover, we determined whether SIRT6 is associated with PPARα exposure of cells to RV by IP-WB analysis. We cotransfected 293 T cells with flag-tagged SIRT6, treated them with RV, and performed IP-WB analysis. IP with αFlag followed by αPPARα definitely revealed that SIRT6 associated with PPARα upon RV treatment (Fig. 6b, top panel). Moreover, a reciprocal experiment, in which IP was performed using αPPARα followed by WB analysis using αFlag (Fig. 6b, bottom panel), detected the interaction of SIRT6 and PPARα in RV treated cells. RV-mediated SIRT6 expression depends on PPARα (Supplementary Fig. S4).

## Discussion

Recent studies have shown that the insertion of human L1 into genes occurs in the germline during early development, and in somatic cells such as cancer cells^38^. Active human L1 elements are considered an endogenous mutagen; moreover, L1-RTP is induced in many diseases^39^. In particular, exhaustive genome analysis has identified the number of L1 insertions in cancers that develop from epithelial cells^40^. Although the mechanism of L1-RTP induction during carcinogenesis and the other diseases remain elusive, suggested L1-RTP inducers include carcinogens, tryptophan photoproducts, environmental pollutants, and viral protein R (Vpr) a protein encoded by human immunodeficiency virus type-1 (HIV-1)^14–17,41^. We searched among the compounds that regulate L1-RTP but were unable to find compounds that inhibit L1-RTP. This study found that RV inhibited L1-RTP in HeLa cells and HuH-7 cells and investigated the molecular mechanism involved. We discovered that RV, a polyphenol with antioxidant properties, inhibited L1-RTP at concentrations ranging from 5 to 20 μM, which is one-half of the vasodilator effect of RV (30–40 μM)^42^. Under physiological conditions, RV is described as being present at the order of a few μM, so RV-based inhibition of L1-RTP could occur within an in vivo range.

We previously showed that environmental carcinogens, tryptophan photoproducts, and HCAs, originating from the cooking of red meat, induced L1-RTP^14–17^. As these compounds exist within our surroundings, we are frequently exposed to them. RV can reportedly regulate L1 methylation^29^, whereas the mechanism by which RV regulates the metastatic potential of L1 remains elusive. Therefore, we investigated whether RV inhibits compounds that induce L1-RTP and clarified the analysis of the mechanism. We found that RV inhibited FICZ-, MeIQx-, and PhIP-induced L1-RTP in HeLa cells and HuH-7 cells. RV inhibited the phosphorylation of p38 and CREB by FICZ and HCA. Moreover, FICZ- or HCA-induced chromatin recruitment of ORF1 was blocked by RV. Taken together, our data suggested that RV inhibited L1-RTP by inhibiting p38 and CREB phosphorylation as well as by blocking the chromatin recruitment of the L1-ORF1 protein. Recently it was reported that L1 activity dependent on and integrated with cellular phosphorylation regulatory cascades significantly increased our understanding of interactions between L1 and the host^43,44^. We suggested that L1-RTP required MAPK phosphorylation and its downstream transcription factor in previous articles. Several articles reported that RV is a MAPK inhibitor; thus, RV inhibits the L1-RTP inducer. Collectively, L1-RTP required phosphorylation of ORF1 protein and downstream cascade factor or environmental transcription factor in host cells.

To determine the molecular mechanism of RV-mediated L1-RTP inhibition, we examined whether RV-based inhibition of L1-RTP depended on its antioxidant effect. The effect of RV was compared to that of NAC, which was found not to inhibit L1-RTP; thus, RV acts independently of its antioxidant effect. We further tested this using an inhibitor and siRNA-based depletion of the PPARα, the first target factor identified for RV. Interestingly, biochemical analysis revealed that RV inhibited L1-RTP in a PPARα-dependent manner. The 4’-hydroxyl group of resveratrol is critical for the direct activation of PPARα^45^; thus, we suggested that RV first interacts with and binds to PPARα to initiate the L1-RTP inhibition step. PPARα inhibitors reportedly induce L1 activation^37^, suggesting that PPARα is involved in the pathway that induces L1-RTP.

Many studies have recently identified potential restriction factors for L1^33^. SIRT6 was found to repress L1 activity by ribosylating Kruppel-associated box (KRAB)-associated protein 1 (KAP1) of transcription inhibition factor. SIRT6 binds to the 5ʹ-UTR of the L1 promoter and ribosylates KAP1^25^. The SIRT6-mediated KAP1 mono-ADP-ribosylation (MARylation) promotes the interaction between KAP1 and SIRT6, thereby promoting the recruitment to the L1 5ʹ-UTR^24^. L1-RTP is facilitated by the chemical modification of the Poly (ADP-ribose) Polymerase 2 recruitment of replication protein A to other DNA repair proteins. Moreover, RV was shown to increase SIRT genes’ expression. Here, we confirmed that RV increased SIRT6 protein expression. Combined with the finding that SIRT6 interacts with L1, this strongly suggests that it mediates inhibition of L1-RTP. The cell signal is PPARα as target factor and involves the regulatory function of SIRT6 and L1. SNF2H is involved in RV-mediated inhibition of L1-RTP, but SNF2H did not interact with ORF1 by IP-WB. We confirmed that expression of SIRT6 increased by RV is inhibited by PPARα and SNF2H siRNA.

**Figure 7:**
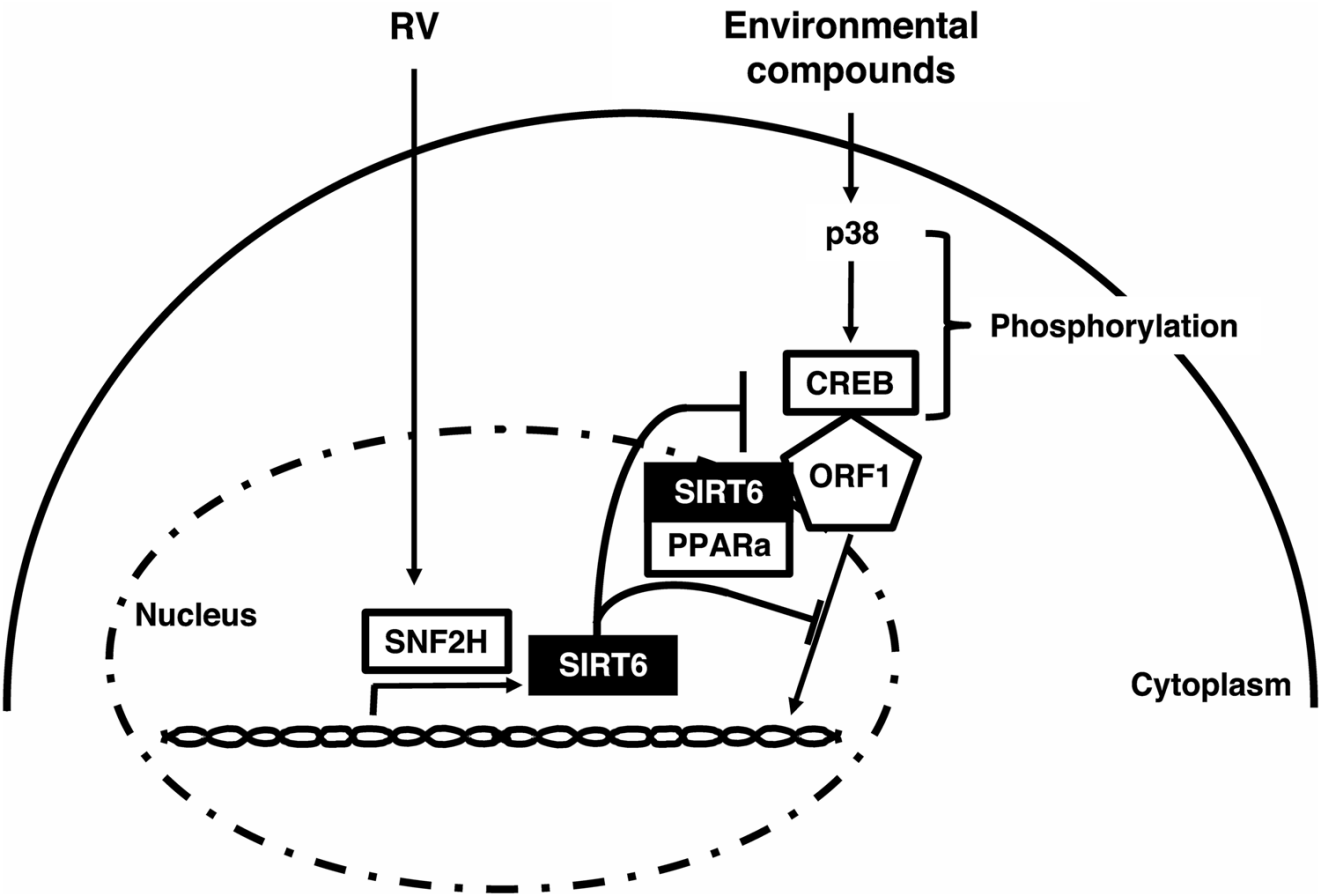
Hypothetical modes of inhibition of L1-RTP by RV. RV induces the transcriptional activation of SIRT6 in a manner depending on SNF2H. Additionally, RV promotes molecular interaction of PPARg, SIRT6, and ORF1 and blocks chromatin recruitment of ORF1 that requires CREB phosphorylated by environmental compounds via p38.

In our current study, we showed that RV directly modulates the PPARα receptor to inhibit L1-RTP. Furthermore, we suggest a new signaling pathway in which SIRT6 interacts with p38 in MAPK signaling, with RV inhibiting L1-RTP via suppression of MAPK signaling. Cea et al. reported that SIRT6 binds DNA damage sites and recruits and blocks MAPK signaling^46^. We suggested that RV inhibiting L1-RTP by two cell signaling pathways. First, RV directly modulated the PPARα receptor and SNF2H upregulated SIRT6 expression inhibited L1; second, ORF1 interacted with SIRT6 and PPARα blocked MAPK signaling (Fig. 7). SIRT1 and SIRT7 are involved in RV-induced suppression of L1-RTP, but they did not bind to ORF1 (Supplementary Fig. S3). These findings suggest that RV inhibits the genomic instability caused by L1-RTP, altering the human cells’ genome. There is a low frequency of L1-RTP resulting from induction with FICZ and HCAs (approximately one in every 10^4^ cells), compounds that induce autoimmune diseases and cancer development. The effect of RV is essential from the perspective of preventive medicine—we are exposed to ultraviolet light and carcinogens in the environment regularly. We reported previously that the picomolar levels of FICZ (3 pM) could induce L1-RTP^14^; notably, about 8 pM FICZ is generated after 24-h exposure of tissue-culture medium to ordinary laboratory light^47^. Moreover, picomolar levels of HCAs induced L1-RTP, comparable to the PhIP concentration detected in human breast milk^48^. These data suggest that somatic cells are exposed to factors that promote genome instability in the typical life environment. As people age, genetic instability is stimulated by exposure to environmental and intrinsic factors. Activation of transposable elements occurs due to environmental factors and can extend to loss of neuronal function^49,50^.

Sirtuins protect against many of the various aging-associated conditions, including skin damaged by UV radiation of SIRT1 in human keratinocytes^51^. RV can reverse this process in a SIRT1 dependent manner. SIRT7 mediated H3K18 deacetylation regulates the L1 expression and promotes L1 association with elements of the nuclear lamina^26^. As SIRT6 plays a crucial role in silencing L1 activity, SIRT6 activator as RV may prove effective at counteracting the age-related loss of L1 silencing. Our results demonstrate that SIRT1, SIRT6, and SIRT7 were associated with the L1 element and PPARα, so RV inhibited genome instability, including L1. RV appears to promote genomic stability, limiting the occurrence of genetic mutations.

In this study, RV-mediated inhibition of L1-RTP depended on PPARα, SIRT1, 6, 7, and SNF2H. RV inhibits p38 and CREB phosphorylation, which are involved in MAPK signaling, and chromatin recruitment of the L1-ORF1 protein. Furthermore, RV increased the expression of SIRT6, which inhibited L1 activation. We suggest that RV directly modulates PPARα-mediated L1-RTP in somatic cells and that MAPK signaling closely interacts with SIRT6 preventing human diseases, such as cancer and sporadic cases of inborn errors.

## Methods

### Chemicals and cells

Protease and phosphatase inhibitors were purchased from Roche Diagnostics (Basel, Switzerland). Resveratrol and *N*-acetylcysteine (NAC) were procured from Wako Pure Chemical Industries, Ltd (Osaka, Japan). Acetoxy-forms of HCAs (PhIP: molecular weight 286.3; MeIQx: molecular weight 275.2) were synthesized (NARD Institute, Amagasaki, Hyogo, Japan). FICZ is a tryptophan photoproduct (Enzo Life Sciences, NY, USA). GW6471 PPARα inhibitor was purchased from Wako (Osaka, Japan). Neomycin (Wako) and hygromycin (Hygro, Sigma, MO, USA) were used after checking the cytotoxic effects in each cell line. Antibodies against PPARα (Abcam, Tokyo, Japan), sirtuin 1 (SIRT1), sirtuin 3 (SIRT3), sirtuin 6 (SIRT6), sirtuin 7 (SIRT7), p38, phosphorylated p38, CREB, phosphorylated CREB, and SNF2H (Cell Signal Technology, Tokyo, Japan), H2AX (Millipore), enhanced green fluorescent protein (MBL, Nagoya, Japan), Anti-FLAG Tag M2 antibody (Sigma MO, USA), and glyceraldehyde 3-phosphate dehydrogenase (GAPDH) (Trevigen, Gaithersburg, MD, USA) were used as primary antibodies. A rabbit polyclonal antibody against human ORF1 was generated using the peptide MGKKQNRKTGNSKTQSAC as an immunogen (Medical and Biological Laboratories)^14^. Anti-mouse and anti-rabbit secondary IgG conjugated to horseradish peroxidase were obtained from DAKO Japan (Tokyo, Japan).

### L1 retrotransposition

*L1* retrotransposition was assayed as described previously^14–19,52,53^ using reporter vectors based on pCEP4/L1 mneoI/ColE1 (pL1-Neo^R^). The construct contained all components of human *L1* in a single transcriptional unit, with a neomycin-resistance gene in the reverse orientation. When *L1* retrotransposes, the intron within the neomycin (G418, Wako) resistance gene is spliced out, resulting in a neomycin-resistant cell. Constructs were transfected into ~ 2.0 × 10^6^ cells using Lipofectamine 2000 (Life Technologies), with selection for 3 days on 25 μg/mL hygromycin. Transformants were then transferred to 100-mm plates at ~ 1 × 10^5^ per six plates, treated for an additional 2 days with the indicated amounts of chemicals, and finally grown on 300 (HeLa cell) or 250 (HuH-7 cell) μg/mL neomycin to select for cells in which retrotransposition occurred. After 2–3 weeks, colonies were stained with methylene blue and counted. Plating efficiency was examined to exclude cytotoxic effects of RV and observed that uM of RV. HeLa cells were treated for 2 days with RV at doses ranging from 200 to 1.25 μM, and 500 cells of each test group were plated onto 6-cm plates. After additional 7 days, cells were fixed and stained with methylene blue.

### Western blotting

WB was analyzed as described previously^14–19^. Cells were washed with phosphate-buffered saline and resuspended in 50 mM Tris–HCl pH 7.6, 150 mM NaCl, 1 mM EDTA, 0.1% SDS, 0.5% deoxycholic acid, 1% NP-40, and protease inhibitors. Cells were ultrasonicated for 12.5 min (10 s on, 20 s off) at medium power (250 W) at 4 °C with a Bioruptor (UCD-250; Cosmo Bio, Tokyo, Japan), and soluble cellular extracts were recovered after centrifugation for 10 min at 16,000×*g*. Protein concentration was determined using the BCA Protein Assay Reagent Kit (Thermo Scientific Waltham, MA USA), and extracts were analyzed by WB. Blots were probed with primary antibodies, labeled with secondary antibodies conjugated to horseradish peroxidase, and visualized using Pierce Western Blotting Substrate Plus (Thermo Scientific). The original data obtained using WB and the result of adjusting the brightness using Photoshop are shown in the supplementary information.

### RNA-interference experiments

Two small interfering RNAs (siRNAs) were prepared (Thermo Scientific), and their functions were evaluated by transfection into cells followed by WB analysis^14,15^. We transfected 10 nM of siRNA using Lipofectamine RNAiMAX in HeLa cells and HuH-7 in 2.0 × 10^6^ cells. The nucleotide sequences of each siRNA are shown in Table 1. Each siRNA was introduced on day 3 after initial transfection with pL1-Neo^R^ to evaluate the inhibitory effects of the siRNAs on L1-RTP induction. Two days later, the cells were replated, incubated for 2 days with RV, and analyzed. As a control, Silencer Negative Control siRNAs (Thermo Scientific, Cat# AM4613) were used.

### Chromatin recruitment of ORF1

We used the pORF1-TAP (tandem affinity purification) construct^14,15^, which encodes a chimeric protein of ORF1 protein A and the calmodulin-binding protein. On day 2, after transfection of pORF1-TAP into HeLa cells, FICZ or HCAs was added to the culture medium before RV, and cell extracts were prepared on the following day. The chromatin-enriched fraction (chromatin fraction) was isolated using the Subcellular Protein Fractionation Kit (Thermo Fisher Scientific) with micrococcal nuclease as described previously^14,15^. Detection of ORF1-TAP was performed by probing with a horseradish peroxidase-conjugated α-rabbit IgG antibody. H2AX served as a positive control for the chromatin fraction. Fractionation and detection of H2AX were evaluated using previously described methods^15^. A subcellular fractionation analysis revealed ORF1 after transfection of expression vectors encoding a chimeric ORF1-TAP protein.

### Immunoprecipitation assay

Immunoprecipitation (IP) assay was carried out, according to the reported method^14,15^. HEK293T cells were transfected with each plasmid by Lipofectamine 2000. To express ORF1-EGFP, flag-tagged SIRT6 flag-tagged ovalbumin (OVA), and ST (streptavidin-tagged)-EGFP, we constructed pORF1-EGPF, pFlag-SIRT6, pFlag-OVA, and pST-EGFP. Cells were suspended in IP buffer composed of 20 mM Tris–HCl (pH 7.6), 5 mM EDTA, 150 mM NaCl, 0.5% NP-40, 10% glycerol. 500 μg of cell extract were reacted with 4 μg of αFlag or EGFP or αPPARα and then recovered with Dynabeads protein G beads (Thermo scientific). About one-tenth of each extract subjected to IP was loaded onto SDS-PAGE and simultaneously analyzed as an input sample. The immunohistochemical analysis was done by the reported method^14,15^. We used the TrueBlot (Rockland Immunochemicals, Inc.) secondary antibody, which masks the detection of the heavy and light IgG chains, and thus, it displayed them as thin or undetectable bands.

### Statistical analysis

Statistical significance was evaluated using the Mann–Whitney *U*-test, with an n value of more than four. *P*-values of less than 0.05 were considered statistically significant.

## Supplementary Information

Supplementary Figures.

## Data Availability

The data that support the findings of this study are available from the corresponding author upon reasonable request.
