# Supplementary material for: Resveratrol blocks retrotransposition of LINE-1 through PPAR α and sirtuin-6
Source: Sci Rep. 2022 May 11;12:7772. doi: 10.1038/s41598-022-11761-0 (PMC9095727; doi:10.1038/s41598-022-11761-0)
Supplement: Supplementary file 1 — Supplementary Figures. [file 41598_2022_11761_MOESM1_ESM.pdf]

Resveratrol blocks retrotransposition of LINE-1 through PPAR α and sirtuin-6

Noriyuki Okudaira[1*], Yukihito Ishizaka[2], Mimi Tamamori-Adachi[1]

[1]Department of Biochemistry, Teikyo University School of Medicine, 2-11-1, Kaga, Itabashi-ku, Tokyo, 173-8605, Japan

[2]Department of Intractable Diseases, National Center for Global Health and Medicine, 1-21-1 Toyama, Shinjuku-ku, Tokyo, 162-8655, Japan

Correspondence to: Noriyuki Okudaira

Department of Biochemistry, Teikyo University School of Medicine

2-11-1, Kaga, Itabashi-ku, Tokyo, 173-8605, Japan

Phone No: +813-3964-3649

Fax No: +813-3964-3649

E-mail: nokudaira@med.teikyo-u.ac.jp

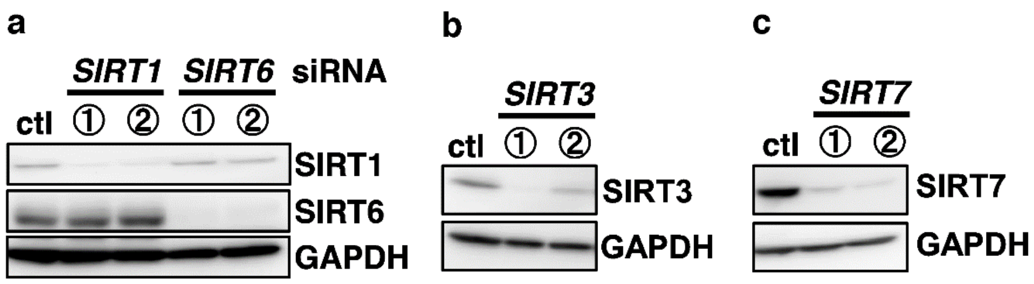

Supplementary Figure S1

a. Effects of SIRT1 and SIRT6 siRNA on the expression of the endogenous SIRT1 and SIRT6 protein. The latter was examined in HeLa cells that had been transfected with pL1-NeoR and siRNA. Ctl, control siRNA (10 nM); ① and ②, SIRT1 siRNA-1 or siRNA-2 (10 nM), ① and ②, SIRT6 siRNA-1 or siRNA-2 (10 nM), respectively.

b, c. Effects of SIRT3 and SIRT7 siRNA on endogenous SIRT3 and SIRT7 protein expression. The latter was examined in HeLa cells that had been transfected with pL1-NeoR and siRNA. Ctl, control siRNA (10 nM); ① and ②, SIRT3 siRNA-1 or siRNA-2 (10 nM), ① and ②, SIRT7 siRNA-1 or siRNA-2 (10 nM).

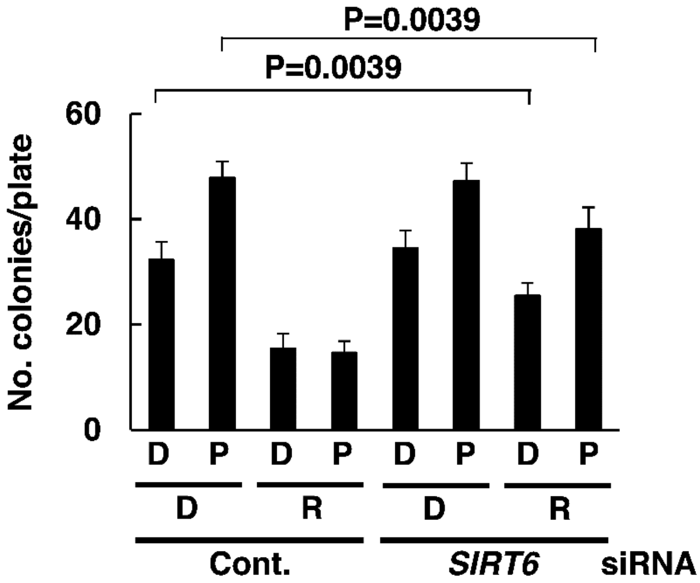

Supplementary Figure S2

Downregulation of SIRT6 dissolves RV inhibited PhIP of L1-RTP. A colony formation assay was performed for HeLa cells after introducing either control siRNA or *SIRT6* siRNA-1. 0.02 % DMSO: D, 18 µM PhIP: P, 20 µM RV: R

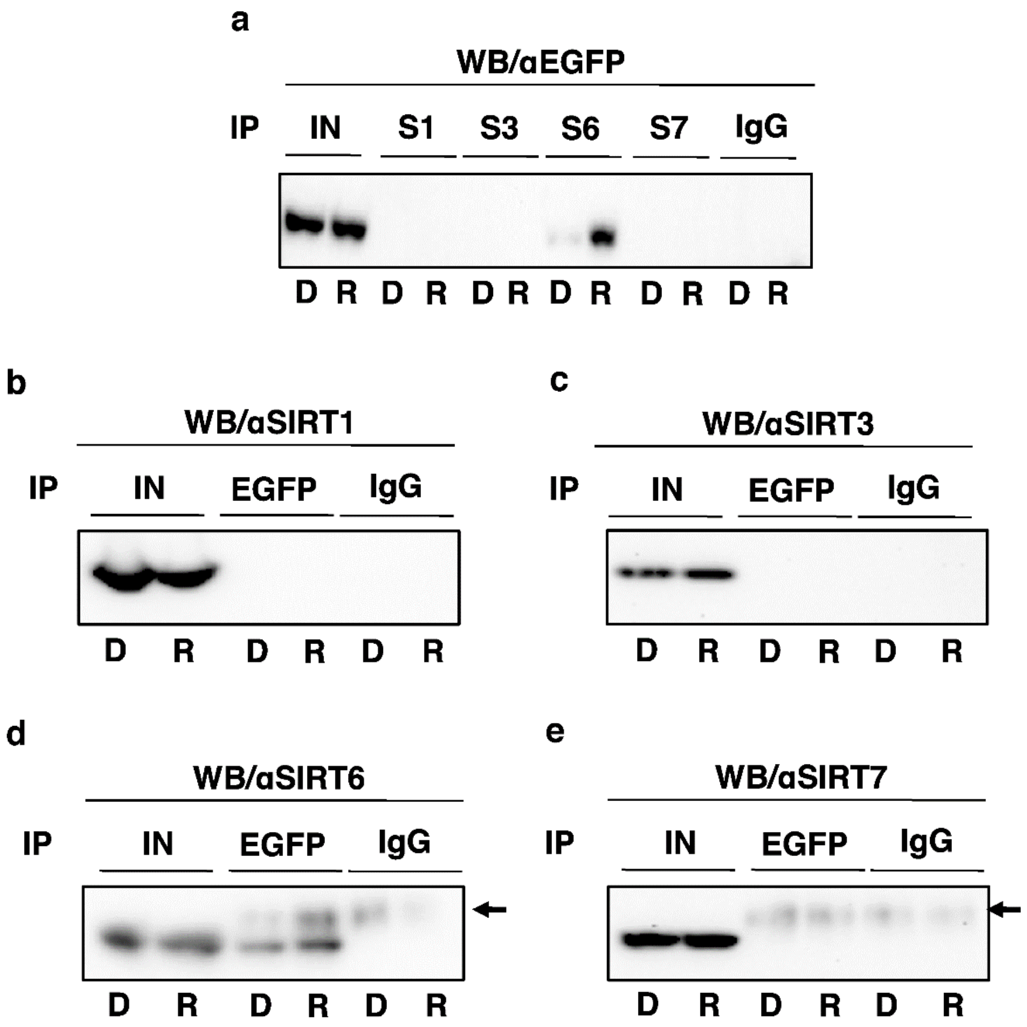

Supplementary Figure S3

RV promotes the association of SIRT6 with L1-ORF1. **a–e**. HeLa cells were transfected with pEGFP-ORF1, and the cell extracts were subjected to IP-WB. **a**. IP of panel was *α*SIRT1, 3, 6, 7 → *α*EGFP. **b**. IP of panel was *α*EGFP → *α*SIRT1. **c**. IP of panel was *α*EGFP → *α*SIRT3. **d**. IP of panel was *α*EGFP → *α*SIRT6. **e**. IP of panel was *α*EGFP → *α*SIRT7. Input, IN; S1, SIRT1; S3, SIRT3; S6, SIRT6; S7, SIRT7. The True blot antibody was the secondary antibody. Arrows indicate the positions of heavy chain. The cells were treated with DMSO (0.02%; D) or RV (20 μM; R).

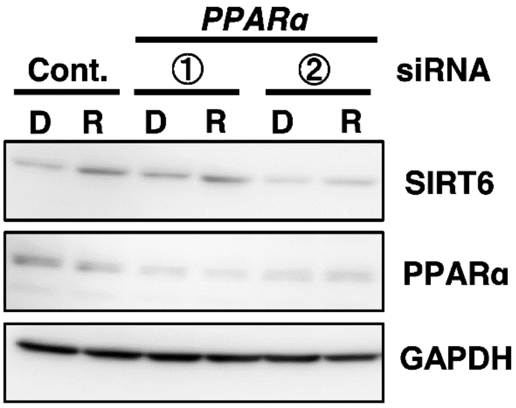

Supplementary Figure S4

RV-mediated SIRT6 expression depends on PPARα. RV treatment increased the level of SIRT6 expression in HeLa cells. WB analysis was performed after introducing either control siRNA or *PPARα* siRNA-1, 2. The cells were treated with 0.02% DMSO (D) or 20 μM RV (R) for 2 days. The relative intensity ± SD is depicted. The effects of *PPARα* siRNAs were significant (p < 0.05). Asterisks indicate statistical significance (p < 0.05 compared to control siRNA in RV treatment).

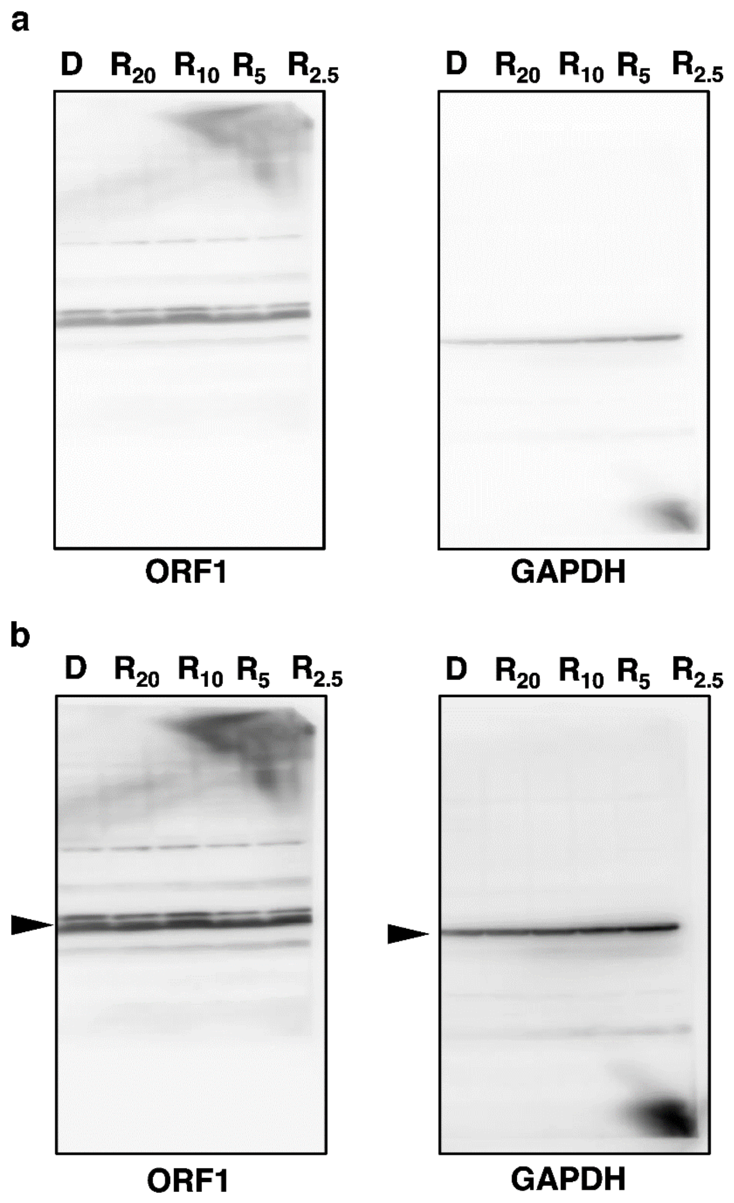

Supplementary Figure S5

Raw data blots (**a**) and normalized blot (**b**) used in Fig. 1G. The arrowhead indicates the band used in the study. The blot image represents the extracted lane of the target sample.

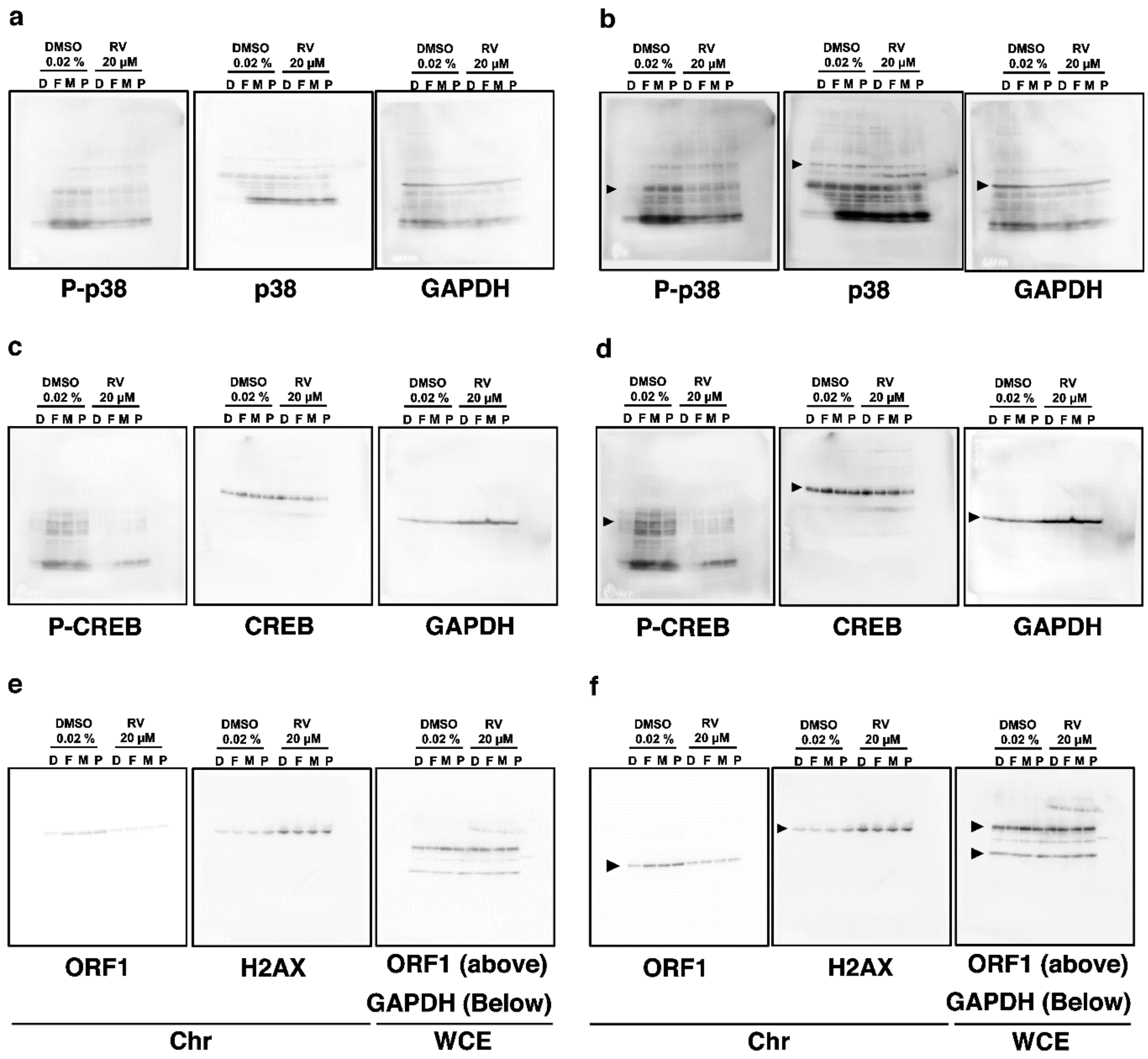

Supplementary Figure S6

Raw data blots (**a, c, e**) and normalized blot (**b, d, f**) used in Fig. 2E (**a, b**), 2F (**c, d**), and 2G (**e, f**). The arrowhead indicates the band used in the study. ORF1 and GAPDH were detected on the same membrane because ORF1 has protein A attached to its C-terminal, which can be detected by IgG (**e** and **f**).

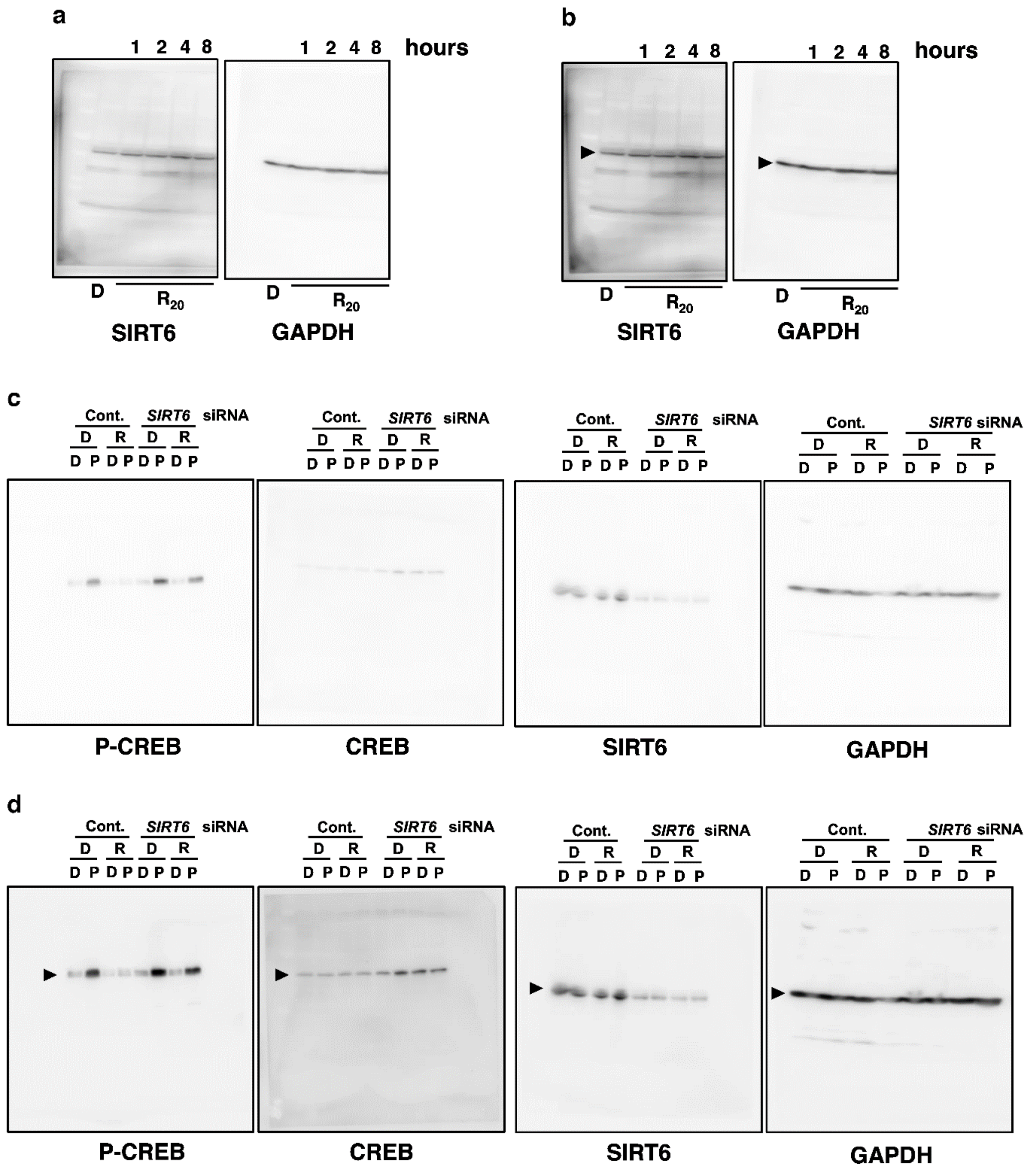

Supplementary Figure S7

Raw data blots (**a, c**) and normalized blot (**b, d**) used in Fig. 3B (**a, b**) and 3C (**c, d**). The arrowhead indicates the band used in the study. The blot image represents the extracted lane of the target samples (**a, b**).

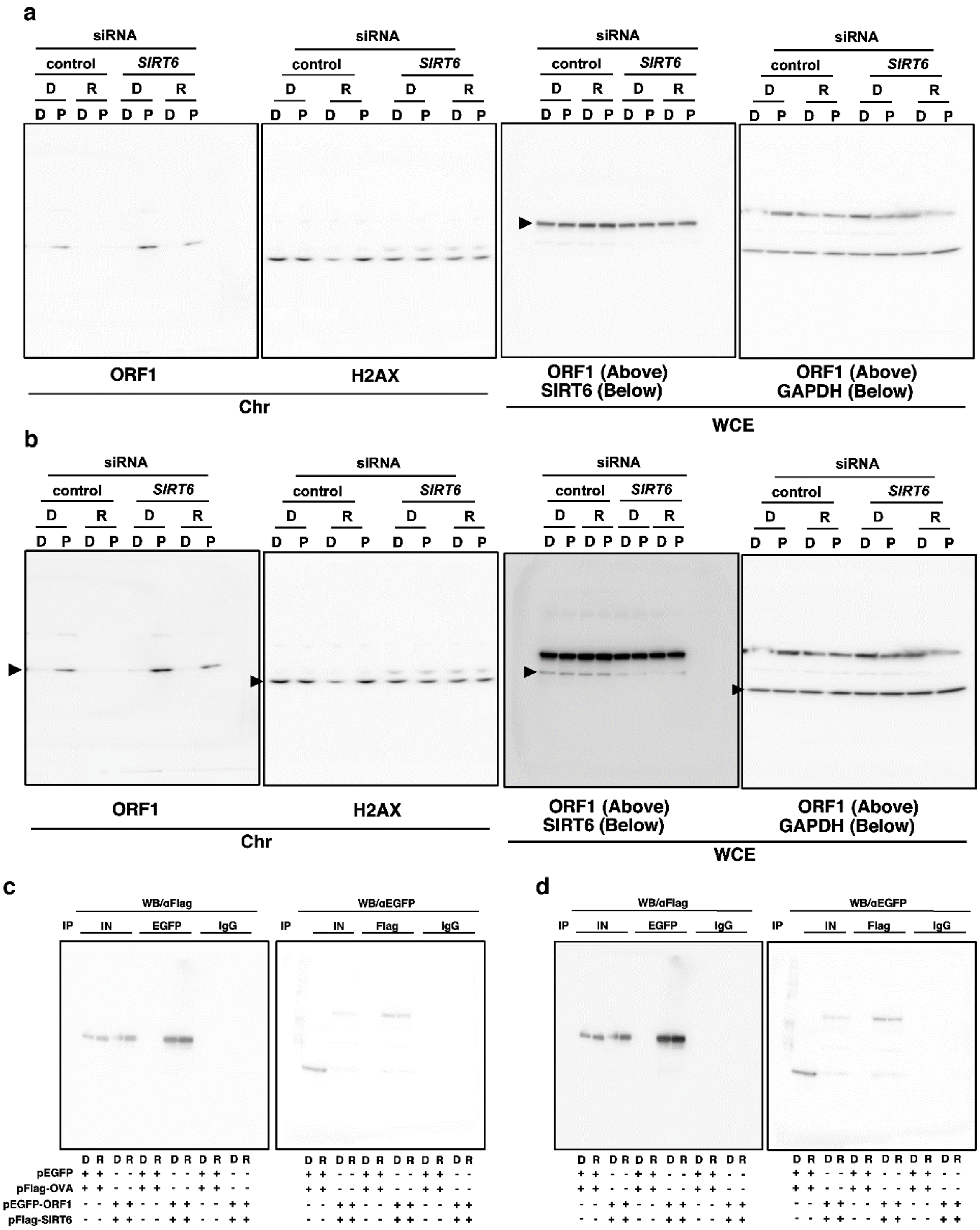

Supplementary Figure S8

Raw data blots (**a, c**) and normalized blot (**b, d**) used in Fig. 3D (**a, b**) and 3E (**c, d**). The arrowhead indicates the band used in the study. ORF1 and SIRT6 or ORF1 and GAPDH were detected on the same membrane because ORF1 has protein A attached to its C-terminal, which can be detected by IgG (**a, b**). D was used for Figure 3E. The blot image represents the extracted lane of the target samples (**a, b**).

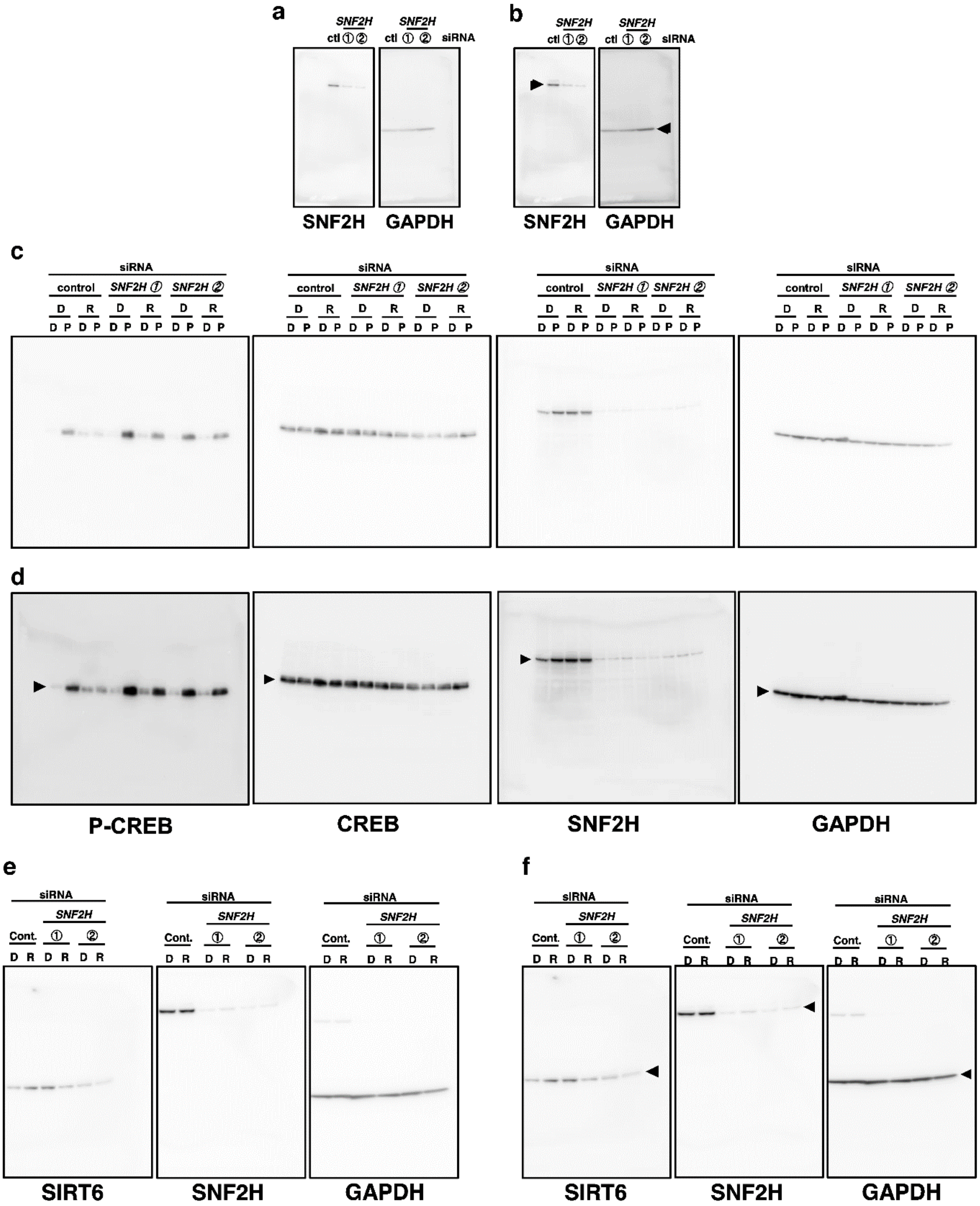

Supplementary Figure S9

Raw data blots (**a, c, e**) and normalized blot (**b, d, f**) used in Fig. 4A (**a, b**), 4C (**c, d**), and 4E (**e, f**).
The arrowhead indicates the band used in the study.The blot image represents the extracted lane of the target samples (**a, b, e** and **f**).

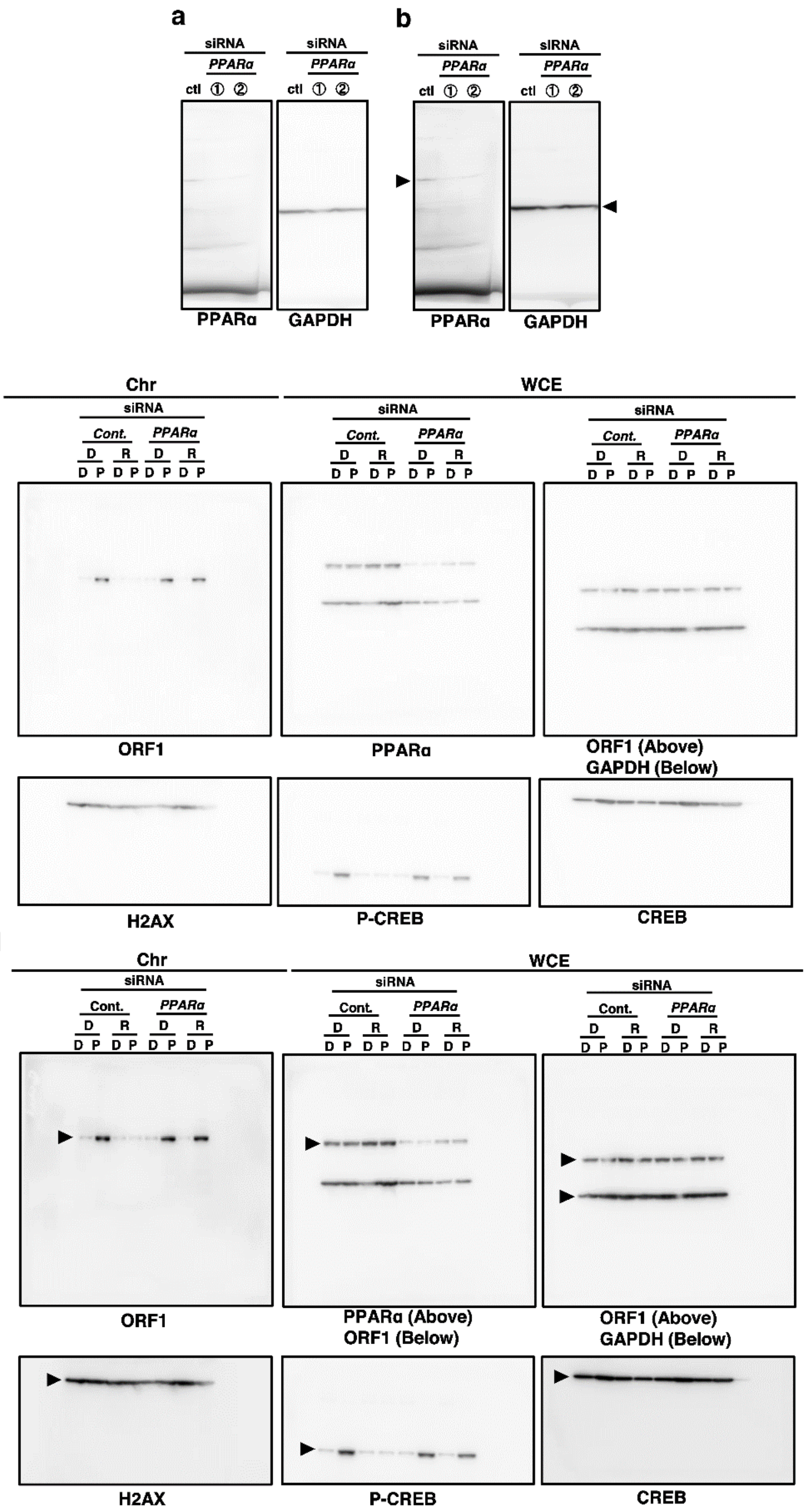

Supplementary Figure S10

Raw data blots (**a, c**) and normalized blot (**b, d**) used in Fig. 5C (**a, b**) and 5E (**c, d**). The arrowhead indicates the band used in the study. The blot image represents the extracted lane of the target samples (**a, b**). PPARα and ORF1 or ORF1 and GAPDH were detected on the same membrane because ORF1 has protein A attached to its C-terminal, which can be detected by IgG (**c, d**). The blots for H2AX, p-CREB, and CREB represent the antibody reaction data after cutting the membrane.

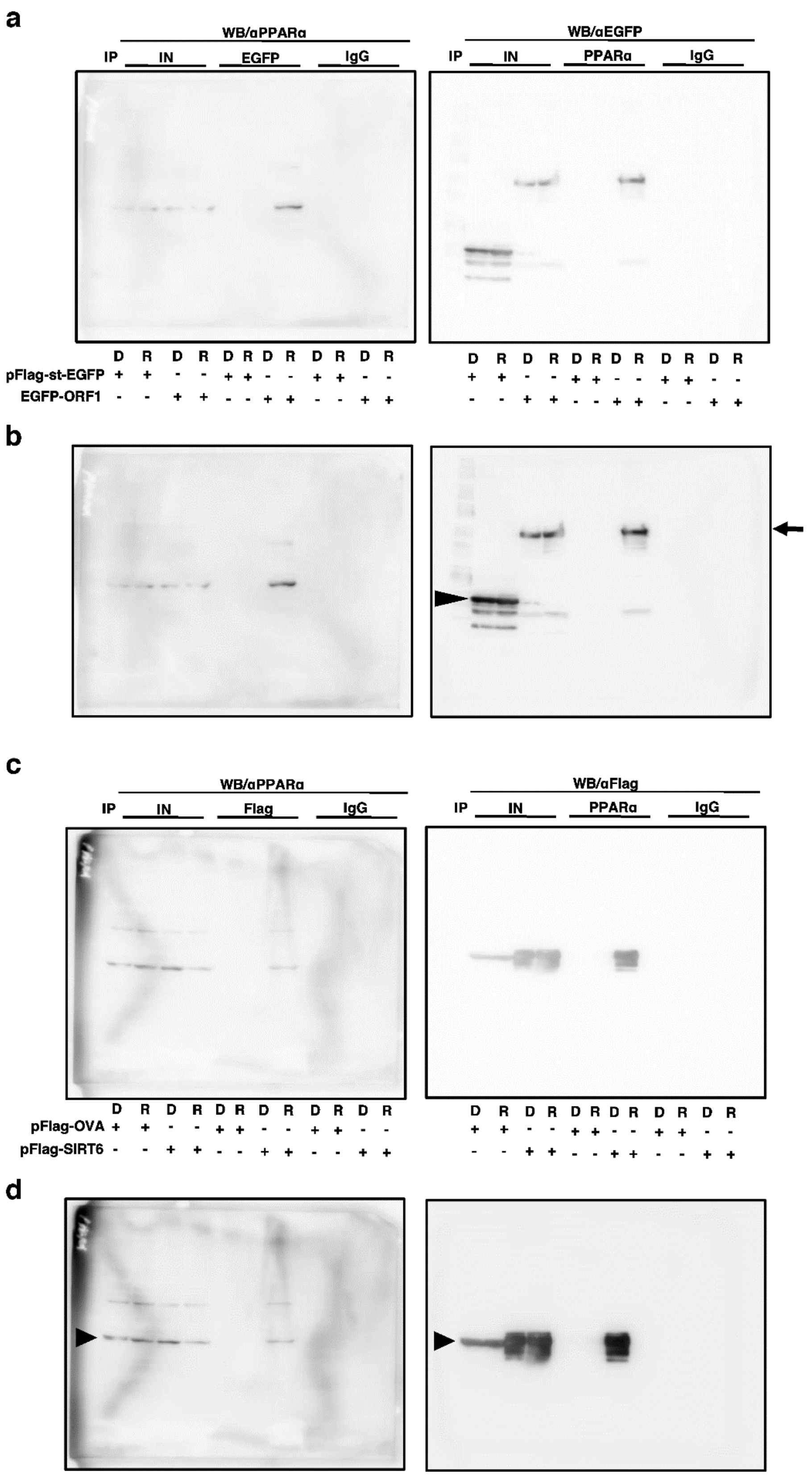

Supplementary Figure S11

Raw data blots (**a, c**) and normalized blot (**b, d**) used in Fig. 6A (**a, b**) and 6B (**c, d**). **b** was used for Figure 6A. **d** was used for Figure 6B. The arrow and arrowhead indicate positions of EGFP-ORF1 and Flag-st-EGFP, respectively.

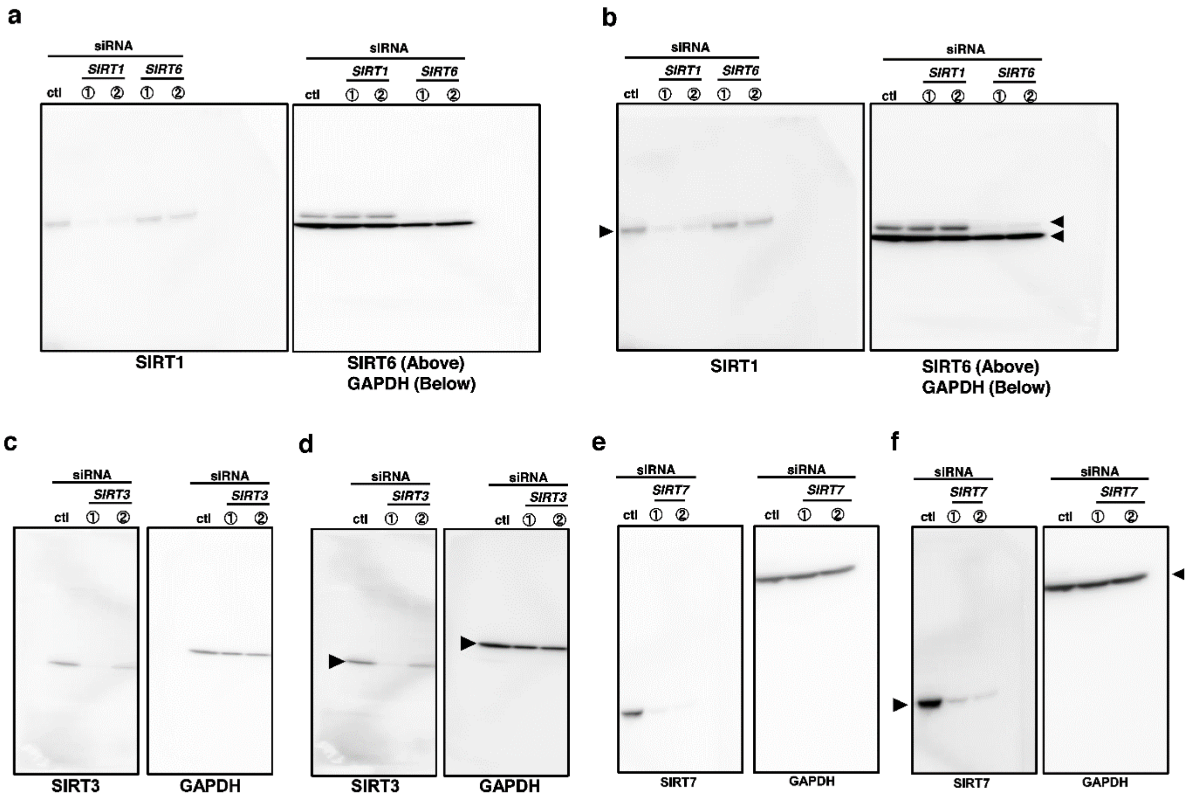

Supplementary Figure S12

Raw data blots (**a, c, e**) and normalized blot (**b, d, f**) used in Supplementary Fig. S1A (**a, b**), S1B (**c, d**), S1C (**e, f**). The arrowhead indicates the band used in the study. The blot image is the extracted lane of the target sample (**a, b, c, d, e, f**). The blots for SIRT1, SIRT6, and GAPDH are the data of antibody reaction after cutting the membrane (**a, b**). The antibody reaction between SIRT6 and GAPDH was performed on the same membrane (**a, b**).

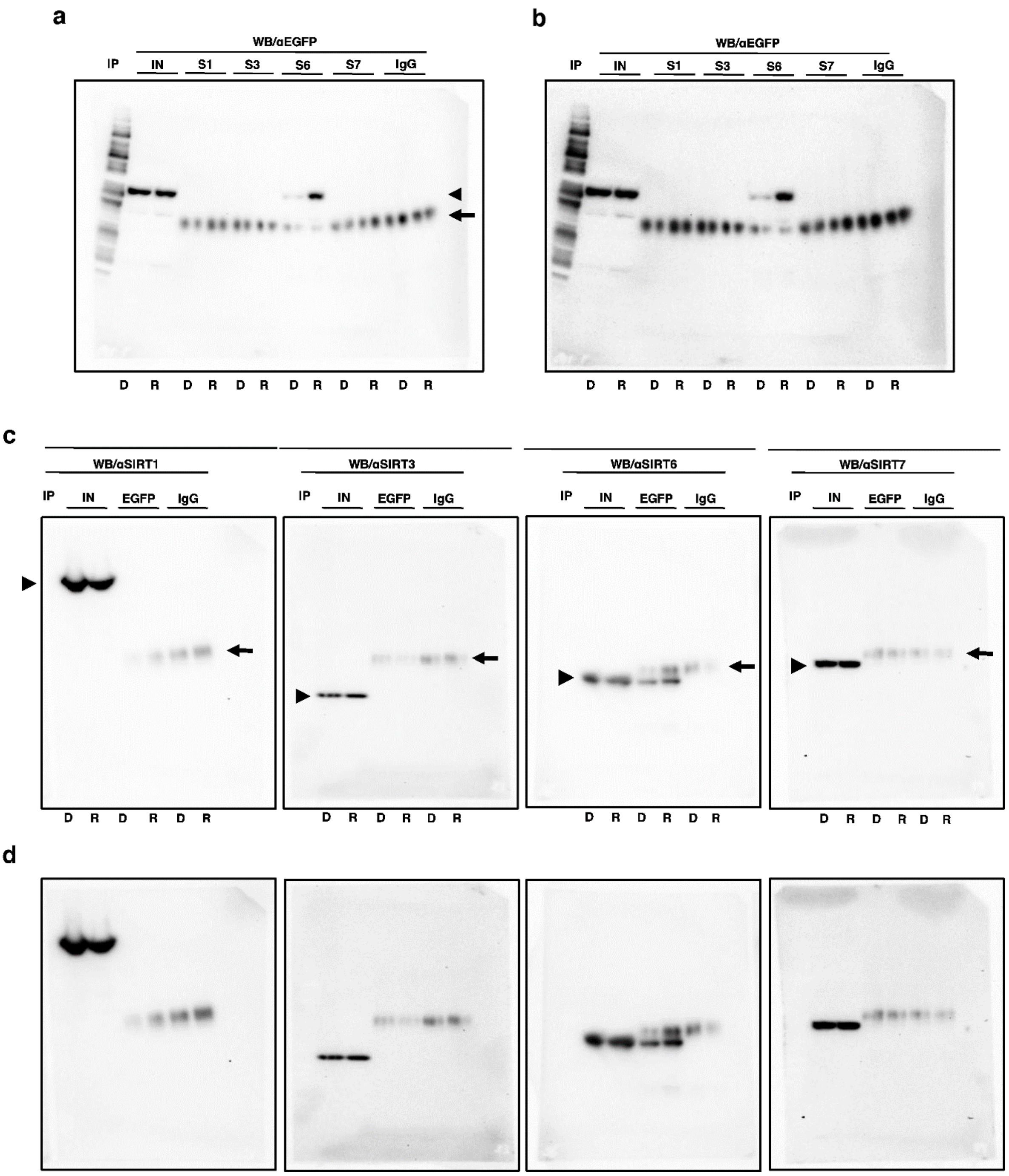

Supplementary Figure S13
Raw data blots (**a, c**) and normalized blot (**b, d**) used in Supplementary Fig. S3. **a** was used for Supplementary Fig. S3A. **c** was used for Supplementary Fig. S3B-E. Arrowhead indicates the band used in the study. The arrow indicates the positions of heavy chain.

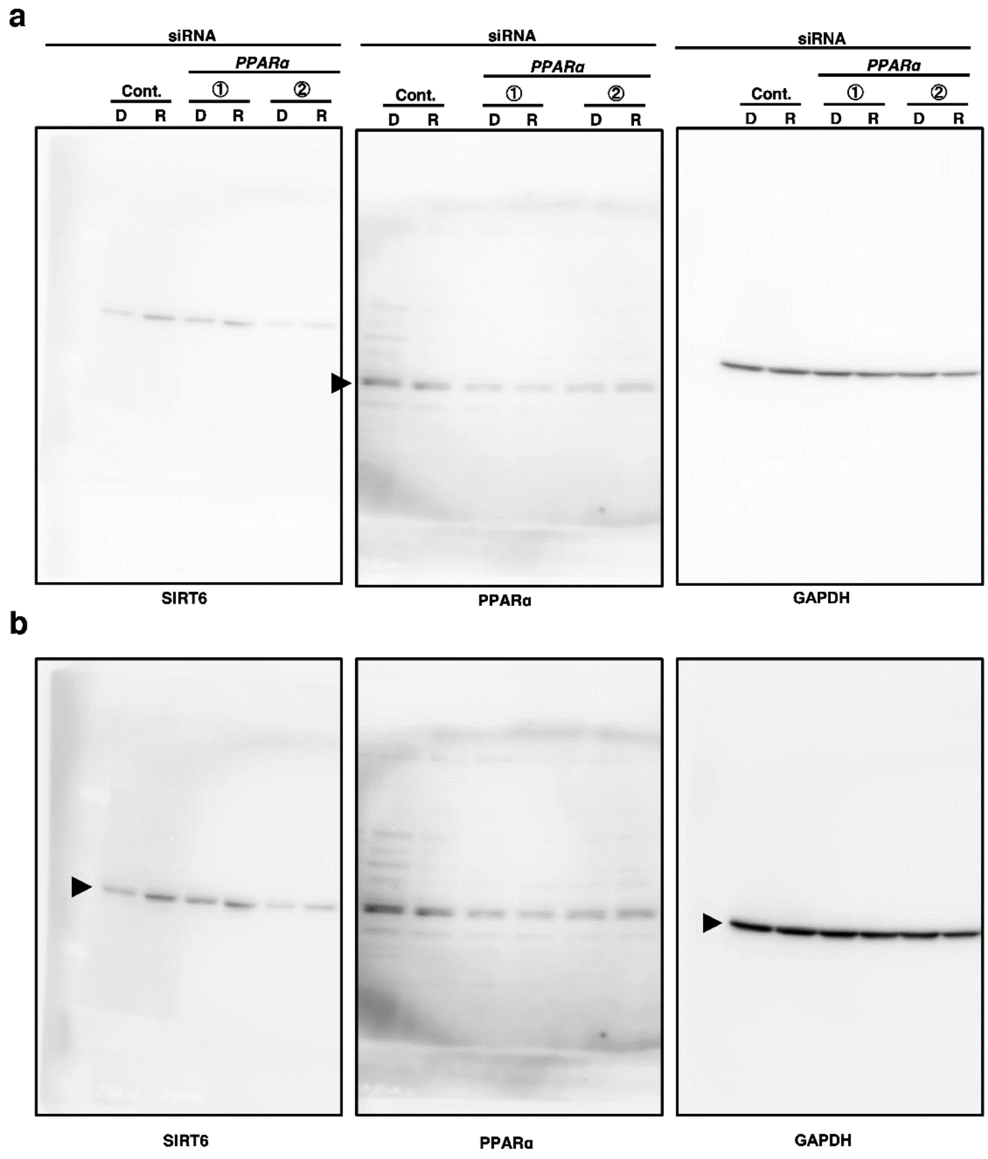

Supplementary Figure S14

Raw data blots (**a**) and normalized blot (**b**) used in Supplementary Fig.S4. The arrowhead indicates the band used in the study. The blot image represents the extracted lane of the target samples (**a, b**).
